# Activation of zinc uptake regulator by zinc binding to three regulatory sites

**DOI:** 10.1093/nar/gkae079

**Published:** 2024-02-13

**Authors:** Yunchan Choi, Junseock Koh, Sun-Shin Cha, Jung-Hye Roe

**Affiliations:** Laboratory of Molecular Microbiology, School of Biological Sciences, College of Natural Science, Seoul National University, Seoul 08826, Republic of Korea; Laboratory of Biophysical Chemistry, School of Biological Sciences, College of Natural Science, Seoul National University, Seoul 08826, Republic of Korea; Protein Research Laboratory, Department of Chemistry and Nanoscience, Ewha Womans University, Seoul 03760, Republic of Korea; Laboratory of Molecular Microbiology, School of Biological Sciences, College of Natural Science, Seoul National University, Seoul 08826, Republic of Korea

## Abstract

Zur is a Fur-family metalloregulator that is widely used to control zinc homeostasis in bacteria. In *Streptomyces coelicolor*, Zur (*Sc*Zur) acts as both a repressor for zinc uptake (*znuA*) gene and an activator for zinc exporter (*zitB*) gene. Previous structural studies revealed three zinc ions specifically bound per *Sc*Zur monomer; a structural one to allow dimeric architecture and two regulatory ones for DNA-binding activity. In this study, we present evidence that Zur contains a fourth specific zinc-binding site with a key histidine residue (H36), widely conserved among actinobacteria, for regulatory function. Biochemical, genetic, and calorimetric data revealed that H36 is critical for hexameric binding of Zur to the *zitB* zurbox and further binding to its upstream region required for full activation. A comprehensive thermodynamic model demonstrated that the DNA-binding affinity of Zur to both *znuA* and *zitB* zurboxes is remarkably enhanced upon saturation of all three regulatory zinc sites. The model also predicts that the strong coupling between zinc binding and DNA binding equilibria of Zur drives a biphasic activation of the *zitB* gene in response to a wide concentration change of zinc. Similar mechanisms may be pertinent to other metalloproteins, expanding their response spectrum through binding multiple regulatory metals.

## Graphical Abstract

**Graphical Abstract:**
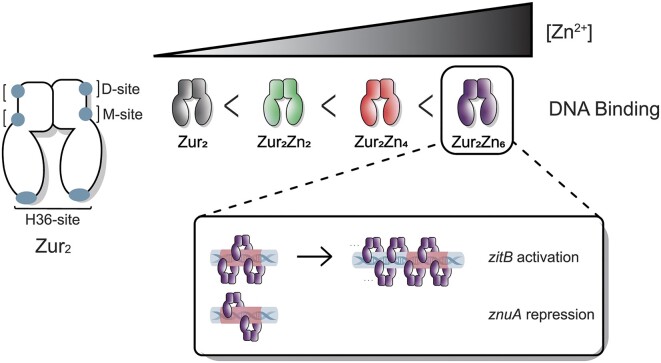

## Introduction

Metal ions are involved in a variety of essential bioenergetics and biochemical processes such as photosynthesis, respiration and nitrogen fixation, and are required for the survival of living organisms (1). Indeed, it has been estimated that one-quarter to one-third of proteins require metal ions for their physiological functions (2–5). To maintain proper amounts of specific metals in the cell, metalloregulators bind to DNA and regulate genes for metal homeostasis, in response to the availability of specific cognate metals (6–9). Zinc is one of the most abundant divalent metals in the cell, mostly bound to proteins and serves catalytic, regulatory, and structural roles (7,10). Though essential, excessive intracellular zinc levels cause cytotoxicity primarily by inhibiting functions of other metalloproteins (11,12). Therefore, living cells constantly maintain zinc homeostasis by coordinated expression of genes involved in zinc acquisition, utilization, trafficking, and efflux pathways, which is modulated by zinc-sensing metalloregulators (8,13).

The zinc uptake regulator (Zur) is a ferric uptake regulator (Fur) family transcriptional factor that regulates the transcription of genes involved in zinc homeostasis in bacteria in response to intracellular bioavailable zinc (14). Since its discovery in *Escherichia coli* and *Bacillus subtilis* (15,16), it has been found in >2600 different species of bacteria (17). It is the most widespread metalloregulator for zinc uptake genes in bacteria. Similar to other Fur family members, zinc-bound Zur predominantly functions as a repressor by binding to a conserved sequence called zurbox, which overlaps the −10 and −35 elements of promoters for zinc uptake (znu) or mobilization genes, and thus blocks the binding of RNA polymerase to their promoters under zinc-replete conditions (14,18,19). In zinc-deficient conditions, apo-Zur has a low DNA binding affinity and dissociates from the zurbox leading to the derepression of target genes. Zn-bound Zur can also function as an activator of genes for zinc exporter, such as *czcD* homologue in *Xanthomonas campestris* (20) and *zitB* in *Streptomyces coelicolor* (21), and *bmtA* gene for zinc-sequestering metallothionein in *Synechococcus* sp. (22).

Zur proteins whose structures have been reported so far exist as a homodimer in solution. They contain two or three zinc-binding sites per monomer, which consists of an N-terminal DNA binding domain (DBD) and a C-terminal dimerization domain (DD) linked by a hinge region (13). Site 1 (C-site) is located in the DD and serves as a structural site that coordinates a zinc ion with four cysteines. Site 2 (M-site) is located in the hinge region and serves as a major regulatory site. Zinc ion binding in site 2 is thought to affect the mutual orientation of the DBD and the DD, which, in turn, regulates DNA binding activity. Zn-binding to site 3 (D-site) has been observed in *M. tuberculosis* (23) and *S. coelicolor* (24). It is located between the hinge and the DD, and its regulatory role to enable graded (expanded) expression of zinc uptake and mobilization genes has been shown in *S. coelicolor* (24). Graded expression of Zur regulon genes has also been observed in *B. subtilis* by sequential binding of zinc ions to site 2 in the dimer (25,26) and in *E. coli* by different DNA binding affinities for different promoters (27). Recently reported Zur from *Synechococcus* does not contain site 2 (M-site), but binds regulatory zinc to the D-site in the active form (22). Structural features of the interaction between Zur and the zurbox DNA have been characterized for *E. coli* Zur (*Ec*Zur) binding to the *znuA* site by X-ray crystallography (27) and *S. coelicolor* Zur (*Sc*Zur) to the *zitB* site by cryo-EM studies (28). *Ec*Zur binds to the *znuA* zurbox as a tetramer (dimer of dimers) while *Sc*Zur binds to the *zitB* zurbox as a hexamer (trimer of dimers).

The role of Zur as a transcriptional activator has been best studied in *S. coelicolor* (21,28). *Sc*Zur regulates zinc homeostasis by responding to a wide range of zinc concentration changes. As bioavailable zinc is below ∼0.2 fM, Zur does not bind to its regulon promoters, allowing expression of zinc-uptake (*znuA*) and zinc-mobilization (*rpmF2*, *rpmG2*) genes (24). As available zinc level increases above ∼ 1 fM, Zur binds to the promoter of its regulon genes, repressing all the negatively-regulated zinc-sparing genes and partially activating a zinc exporter (*zitB*) gene (21,24). As extra zinc is added in the growth medium, *zitB* gets further activated, reaching a maximum level at around 100 μM added zinc. For full activation, the promoter upstream region up to about −140 nt from the transcription start site is required, on which multimeric Zur binding occurs (21). It has been demonstrated that zinc facilitates multimeric binding of Zur on the upstream region of the *zitB* promoter. However, the underlying mechanism by which *Sc*Zur responds to a wide range of zinc concentrations and fully activates the *zitB* expression has remained elusive. In this study, we used a combination of biophysical, biochemical, and genetic analyses to discover a novel zinc-binding site containing a key histidine residue located at the second helical domain of Zur. This site, together with other known regulatory zinc binding sites, significantly contributes to the DNA binding activity of Zur. By constructing a thermodynamic model for coupling between the zinc binding and DNA binding equilibria of Zur, we demonstrate a remarkable increase in DNA binding affinity of Zur upon saturation of all three regulatory zinc binding sites. Such a strong coupling culminates in oligomerization of Zur on the consensus regions of *znuA* and *zitB* DNA as well as on the *zitB* upstream region, and thus the thermodynamic model provides a potential molecular mechanism for the biphasic activation of the *zitB* gene *in vivo*.

## Materials and methods

### Bacterial strains and cultures

*Streptomyces coelicolor* A3(2) M145 strain (the wild type), *Δzur* (29) and *Δzur* with chromosomally integrated *zur* gene (either wild-type or site-specifically mutagenized) expressed from its own promoter were used in this study. All Streptomyces cells were routinely grown in YEME liquid media with 10.3% sucrose (30) starting from spore inoculation. All bacterial strains used in this study are tabulated in Supplementary Information.

### Construction of mutant strains

The plasmid with a promoter and the entire coding region of *S. coelicolor zur* (pSJ703; (24)) was used as a template for PCR. Site-specific mutagenesis was carried out with 9 pairs of mutagenic primers, changing codons for E25, E27, E28, E34, H36, D37, H41, D44 and D60 to alanines. The mutated PCR products were confirmed by DNA sequencing and each product was introduced into pET3a or pSET162 (a derivative of integrative plasmid pSET151) vectors for *in vitro* and *in vivo* studies, respectively. The pSET162-based recombinant plasmids were transferred to *Δzur* mutant of *S. coelicolor* via conjugation. Antibiotic-resistant exconjugants were isolated from single colonies based on standard procedures (30), and verified by nucleotide sequencing of PCR products. All plasmids and oligonucleotides used in this study are tabulated in Supplementary Information (Supplementary Table S5–S7).

### Purification of Zur proteins

The wild-type and variants of Zur (H36A, C79SH87A, H84AE105A) were purified from *E. coli* BL21 (DE3) cells containing pET3a-based recombinant plasmids (Novagen) as previously described with some modifications (21,24,29). Cells were grown in LB medium to an OD_600_ of 0.4, followed by induction with 1 mM isopropyl β-d-thiogalactopyranoside (IPTG) for 1 h at 37°C Harvested cell pellets were resuspended in the binding buffer (20 mM Tris–HCl, pH 7.8, 500 mM NaCl and 5 mM imidazole) and lysed by Emulsiflex (Avestin). The cell lysates were subjected to Ni-NTA column (Qiagen) and proteins were eluted with the elution buffer (20 mM Tris–HCl, pH 7.8, 500 mM NaCl) with imidazole gradient from 25 to 65 mM. The pooled fractions containing Zur proteins were then subjected to affinity chromatography on HiTrap Heparin HP (GE Healthcare), and were eluted with NaCl gradient from 0.1 to 1 M in 20 mM Tris–HCl, pH 7.8. The eluted fractions were finally subjected to size exclusion chromatography on a HiLoad 26/600 Superdex 200 pg (Amersham Biosciences), and eluted with the buffer containing 20 mM Tris–HCl, pH 7.8, 500 mM NaCl, and 5 mM DTT. For EMSA and DNase I footprinting assays, apo-Zur proteins were obtained by dialyzing eluted fractions of purified Zur against buffer A1 (20 mM Tris–HCl, pH 7.8, 250 mM NaCl, 5% glycerol, 5 mM DTT, and 5 mM EDTA) for 12 h at 4°C and then against buffer A2 (20 mM Tris–HCl, pH 7.8, 150 mM NaCl, 10% glycerol and 5 mM DTT) for 12 h at 4°C. For storage, proteins were dialyzed against storage buffer (20 mM Tris–HCl, pH 7.8, 150 mM NaCl, 30% glycerol and 5 mM DTT) for over 16 h at 4°C. For ITC experiment, apo-Zur samples were obtained by dialyzing purified Zur against buffer B1 (20 mM Tris–HCl or HEPES–NaOH, pH 7.8, 250 mM NaCl and 5 mM EDTA) for 12 h at 4°C and then against buffer B2 (20 mM Tris–HCl or HEPES–NaOH, pH 7.8, 250 mM NaCl, 0.5 mM TCEP) for 12 h at 4°C.

### ICP-MS

The metal concentrations of the wild-type and H36A Zur proteins were measured by ICP-MS (NexION 350D, Perkin-Elmer SCIEX) at The National Center for InterUniversity Research Facilities (NCIRF) at Seoul National University, Korea. Zur protein concentrations were determined from UV absorbance at 280 nm using the predicted extinction coefficient (ϵ_280_ = 10 220 M^−1^ cm^−1^).

### RNA analyses

Total RNAs were isolated from *S. coelicolor* A3(2) M145 (wild-type) and Δ*zur* mutant cells grown to OD_600_ of 0.4–0.5 in YEME medium, treated or non-treated with varying amounts of a zinc chelator, *N,N,N’,N’*-tetrakis(2-pyridinylmethyl)-1,2-ethanediamine (TPEN), or ZnSO_4_ for 1 h. Preparation of DNA probes and S1 mapping and quantification procedures were done as described previously (21). For quantitative reverse transcription-PCR (qRT-PCR), isolated RNAs were treated with Turbo DNA-free kit (Thermo) and RiboLock RNase inhibitor (Thermo) to eliminate contaminated DNAs. cDNA fragments were generated from approximately 1 μg of RNAs using random hexamers and RevertAid Reverse Transcriptase (Thermo), and were mixed with gene-specific primers and TOPreal qPCR2X PreMIX (Enzynomics). Subsequent reactions were carried out in the quantitative real-time PCR machine (Stratagene MX300P, Agilent Technologies) by pre-incubating at 95°C for 15 minutes, followed by 40 cycles of 95°C for 10 s and 72°C for 35 s. Analysis of target gene amplification was done by ΔΔCt method, using 16S rRNA values for normalization.

### DNase I footprinting with capillary electrophoresis

The 267 bp *zitB* promoter region from −228 to + 39 nt relative to transcriptional start site was generated by PCR using a 5′ 6-FAM labeled forward primer as described previously (21). DNA probes (250 ng) were incubated with varying amounts of Zur proteins (0.1, 0.45, 0.9, 1.8 μM) at fixed 50 μM ZnSO_4_ or varying amounts of ZnSO_4_ (10, 15, 20, 25 μM) at fixed 1.35 μM Zur in the binding buffer, and were treated with DNase I as described previously (21). Extracted DNA samples were analyzed by ABI 3730 DNA analyzer (Life Technologies).

### Electrophoretic mobility shift assay (EMSA)

Wild-type or mutant Zur proteins were mixed with a fixed amount (65 nM) of different DNA probes (P*znuA* site 1, P*zitB*) in 20 μl of reaction buffer (20 mM Tris–HCl, pH 7.8, 150 mM NaCl, 5 mM DTT, 0.1 mg/ml BSA, 5% glycerol and 0.1 μg of poly(dI-dC)) as described previously (21). After electrophoresis, gels were visualized by using Amersham Typhoon RGB Biomolecular Imager with Cy2 filters.

### Isothermal titration calorimetry (ITC)

All ITC experiments were performed in MicroCal PEAQ-ITC Automated (Malvern Panalytical) at 25°C with a pre-injection time of 300 s, 19 injections (each 2 μl), an equilibration time of 150 or 300 s, and stirring at 1000 RPM. For the zinc-Zur interaction studies, ZnCl_2_ was dissolved in the binding buffer (20 mM Tris, pH 7.8, 150 mM NaCl and 0.5 mM TCEP) containing 3 mM EGTA. For the Zur-DNA titrations, 150 μM of dimeric Zur containing 4.0 equivalents of zinc ions per dimer was titrated into DNA in the binding buffer (20 mM HEPES, pH 7.8, 150 mM NaCl, and 0.5 mM TCEP). The concentrations of DNA probes in the reaction cell varied, depending on the binding stoichiometry between Zur and DNA probes. Raw data were integrated and corrected for the buffer dilution heat using the MicroCal Origin software to obtain the enthalpy change per mole of added ligand. The normalized titration curve was analyzed by a single-class site model to quantify the binding stoichiometry, affinity, and enthalpy. For zinc-Zur titration experiments, the best-fit binding parameters were further corrected for the competition between Zur and EGTA for zinc binding to determine the intrinsic binding affinity and enthalpy (Supplementary Information S1 and Supplementary Figure S13). At least three independent titrations were performed for all ITC experiments.

### Circular dichroism (CD) spectroscopy

Circular dichroism of apo-Zur in the far-UV region was monitored by using Jasco J-815 spectrometer. Both wild-type and H36A apo-Zur proteins were concentrated at 20 μM in 20 mM Na_2_HPO_4_ (pH 7.8) and 50 mM NaCl. The CD spectra were recorded in 1 mm path length cell (cylindrical water-jacketed quartz cell) from 190 to 250 nm at 25°C. Three consecutive scans from each sample were merged to produce averaged spectra. The recorded spectra were corrected for an instrumental offset and the signal from buffer. The corrected CD spectra were converted to mean residue molar ellipticity (deg cm^2^ mol^−1^) (31).

## Results

### Zur contains three regulatory zinc-binding sites

**Figure 1.**
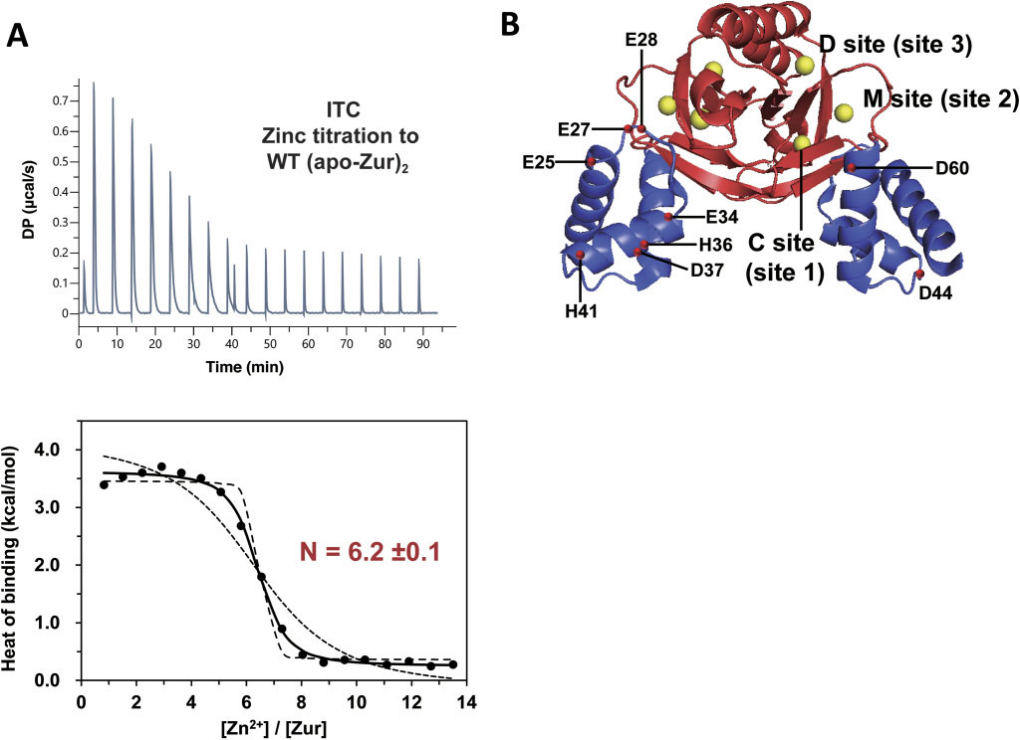
Search for additional zinc binding sites in Zur. (**A**) ITC measurement for the interaction of dimeric apo-Zur with zinc. 2.0 mM ZnCl_2_ was titrated into 24 *μ*M dimeric apo-Zur in the binding buffer containing 20 mM Tris, pH 7.8, 150 mM NaCl, 0.5 mM TCEP and 3.0 mM EGTA at 25°C. The upper panel shows representative ITC heat signals generated in the titration as time traces. The raw heat signals were integrated over time and normalized per mol of zinc injected to obtain heats of binding plotted in the bottom panel as a function of molar ratio. The normalized ITC curve was fitted to a single-class site model (continuous line), yielding the binding stoichiometry, affinity, and enthalpy (Supplementary Table S2). In addition, ITC curves were simulated with the binding constants 10-fold higher (dashed line) and lower (dotted line) than the best-fit value. (**B**) Positions of nine residues for alanine substitution mutation in Zur.

Since Zur responds to a broad range of zinc concentration changes, we initially reasoned that Zur may contain zinc-binding site(s) in addition to the ones previously determined by X-ray crystallography (24). To explore this possibility, we employed isothermal titration calorimetry (ITC), which can accurately detect ligand binding to macromolecules, to assess the stoichiometry of zinc binding to apo-Zur. Assessment of metal binding in apo-Zur by ICP-MS analysis revealed that the average stoichiometry of zinc per purified apo-Zur was 0.96 per monomer, suggesting that only the structural zinc site (site 1 or C-site) is almost completely occupied by zinc in apo-Zur (Supplementary Table S1). Previous studies showed that apo-Zur exists as a dimer bound with structural zinc ions, but without DNA binding activity (24,29). Zinc was titrated into dimeric apo-Zur in the binding buffer (20 mM Tris, 150 mM NaCl, 0.5 mM TCEP) containing 3 mM EGTA, a zinc chelator, to prevent adventitious zinc binding. The normalized ITC curve, with the inflection point of [Zn^2+^]/[Zur dimer] ∼6, demonstrates that an apo-Zur dimer (or monomer) binds 6.0 (or 3.0) equivalents of zinc (Figure 1A). Fitting the ITC curve to a single-class site model yielded the apparent zinc binding affinity of 8.6 (± 1.9) × 10^5^ M^−1^ (or 1.2 ± 0.3 μM in *K*_d_) per site (Supplementary Table S2 and S4), which we corrected for the competition between Zur and EGTA to obtain the intrinsic zinc binding affinity of 2.3 (± 0.5) × 10^13^ M^−1^ (44.2 ± 9.9 fM) (Supplementary Information S1).

The ITC binding isotherms are apparently monophasic and fitted well to the single-class site model (Figure 1A). However, other bacterial Zur and zinc-binding proteins have exhibited heterogeneity or cooperativity among regulatory sites (13,25). Thus, we attempted to estimate the range of potential difference among the zinc binding constants of the three sites in the Zur protomer (*K_i_*, *i*= 1 to 3) by fitting the ITC curve to a three-site model. This analysis showed that the maximum of 20-fold difference between zinc binding constants can be tolerated by the monophasic ITC curve (Supplementary Figure S1). In addition, we performed ITC in HEPES buffer to examine whether the large ionization enthalpy of the original Tris buffer has obscured potential non-monophasic features present in the ITC curve. However, the ITC experiments yielded monophasic binding isotherms in HEPES buffer as well (Supplementary Figure S2), arguing against possible effects of buffer ionization enthalpy on the qualitative feature of the ITC curve. Furthermore, the best-fit binding stoichiometry and affinity (Supplementary Figure S2) agreed well with those quantified in Tris buffer within experimental uncertainty (see Supplementary Figure S2 for further analysis of binding enthalpy).

The 6:1 binding stoichiometry, unambiguously determined by using two different buffer types, indicates that Zur contains an additional zinc-binding site, the third regulatory zinc site, which escaped the previous observation by X-ray crystallography (24) and cryo-EM study (28). To search for this additional zinc-binding site, we created Zur mutants by changing possible zinc-binding residues (glutamate, aspartate, and histidine) in the DNA-binding domain of Zur into alanines (E25A, E27A, E28A, E34A, H36A, D37A, H41A, D44A and D60A), as illustrated in Figure 1B.

### H36A mutation shows defects in activating *zitB*

To investigate the effect of each substitution mutation on the gene regulation activity of Zur *in vivo*, each mutant or the wild type *zur* gene was introduced into the chromosome of *Δzur* strain via the *att* site using integrative pSET162-based recombinant plasmid. The mRNA expression levels of the *znuA* gene encoding a zinc-uptake protein and the *zitB* gene encoding a zinc-exporter were monitored by quantitative reverse transcription-PCR (qRT-PCR), in the wild type (Δ*zur::zur;* WT) or mutant (Δ*zur*::mutant *zur*) cells treated or non-treated with a zinc chelator, TPEN (6 μM), or ZnSO_4_ (100 μM). The wild type M145 strain, M145 with pSET162-derived vector only, and the Δ*zur::zur* strain (WT) all behaved similarly in response to these treatments (Supplementary Figure S3). The changes in the gene expression of *znuA* and *zitB* genes in the WT and *Δzur* strains by TPEN and zinc treatments were consistent with previous observations (21,29).

**Figure 2.**
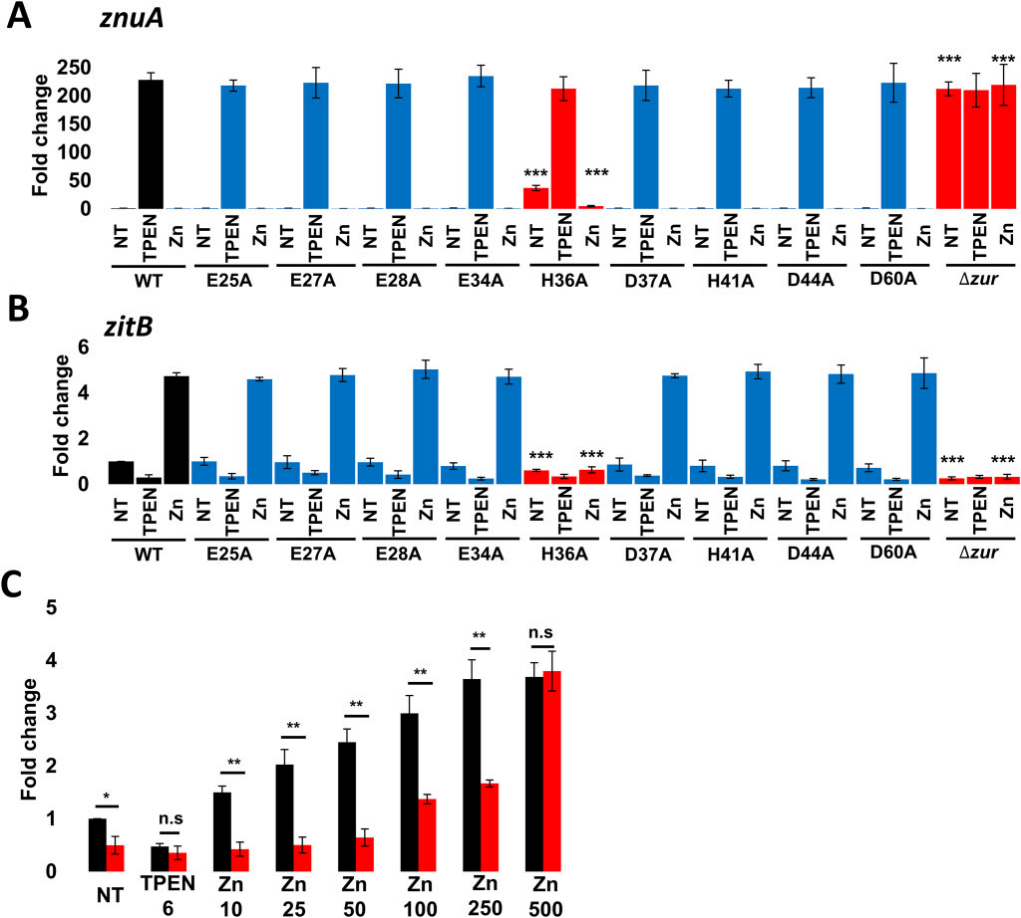
Effect of site-specific mutation of putative metal-coordinating amino acid residues of Zur on the expression of *znuA* and *zitB* genes *in vivo*. (A, B) Effect of 9 alanine substitution mutations of Zur on the transcription of *znuA* (**A**) and *zitB* (**B**) genes. Wild type (Δ*zur::zur*) and mutant (Δ*zur::mutated zur*) cells were treated with a chelator TPEN (6 μM) or ZnSO_4_ (100 μM) for 1 h or none (NT) before cell harvest. RNA levels of *znuA* and *zitB* genes were determined by qRT-PCR. (**C**) Expression of *zitB* under varying concentrations of zinc (10–500 μM) was monitored by S1 mapping in the wild type (black bar) or H36A mutant (red bar). Error bars were presented from three independent experiments. The p-values of less than 0.05 (*), 0.01 (**) or 0.001 (***) by Student's *t*-test were presented.

All of the mutants, except H36A located at the middle of the second alpha helix of Zur, showed similar expression patterns compared with the WT (Figure 2A and B). The basal expression level of the *znuA* gene in H36A mutant was partially derepressed, increased about 37-fold relative to that of the WT strain. However, when treated with 100 μM ZnSO_4_, the *znuA* gene expression was almost fully repressed in the H36A mutant (Figure 2A). On the other hand, the basal *zitB* expression level in the H36A mutant was decreased to about 40% of the WT value, and the gene was not activated by 100 μM ZnSO_4_ (Figure 2B). TPEN treatment in H36A mutant reduced the *zitB* expression to the wild type level. These results indicate that the H36A mutation in Zur caused a partial decrease in its activity as a repressor on *znuA* expression and nearly full loss of activity as an activator on *zitB*.

We further compared the expression profile of the *zitB* genes in the WT and H36A mutant *in vivo* by treating increasing amount of zinc in the medium, followed by S1 mapping analysis of RNA. The *zitB* gene in the WT was activated in a zinc concentration-dependent manner reaching its full expression level in the presence of 250 μM ZnSO_4_. However, the *zitB* expression in H36A mutant was less responsive to zinc, reaching to fully activated level at 500 μM ZnSO_4_ (Figure 2C).

The effect of H36 mutation on cell susceptibility to high zinc was examined by spotting assay. We found that H36A caused cells more susceptible to zinc on plate culture containing 250 or 500 μM ZnSO_4_, compared with the wild type or H41A mutant (Supplementary Figure S4). Even though the effect is less eminent than what was observed for Δ*zur* mutant, the result demonstrates that H36 in Zur does confer cell fitness at high zinc condition.

### H36 constitutes a novel zinc-binding site

**Figure 3.**
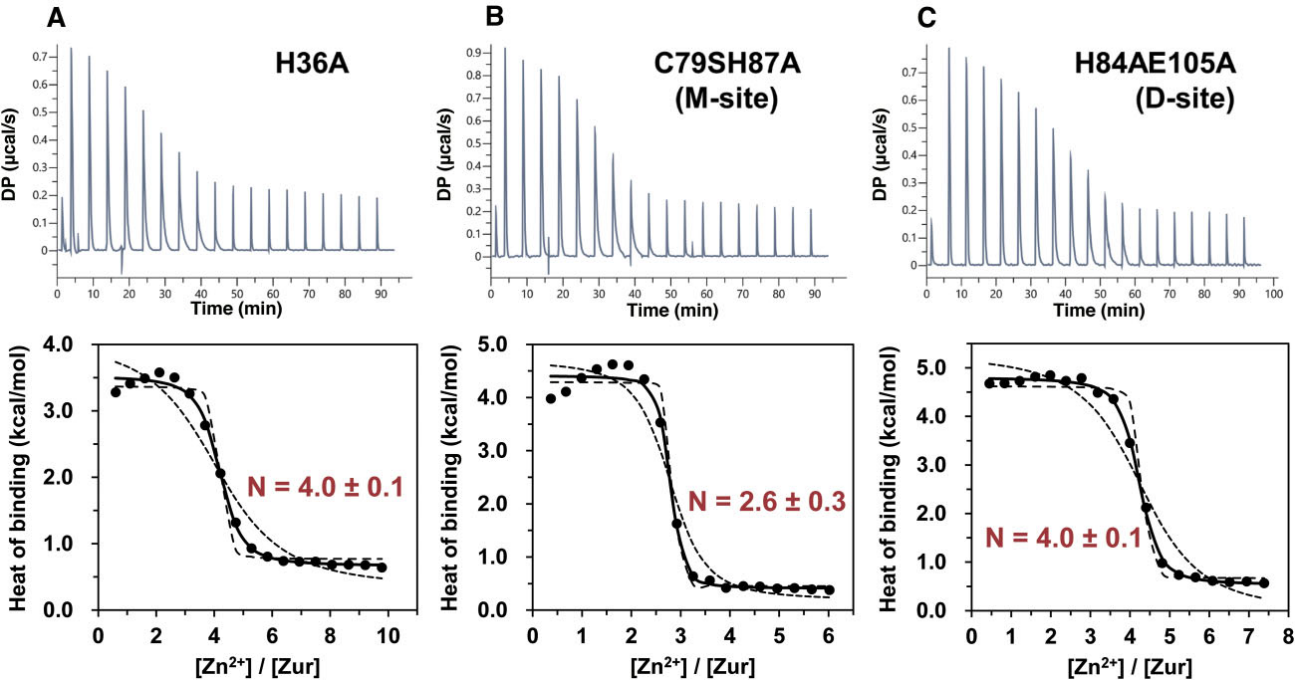
Analysis of zinc binding to Zur mutants by ITC. Zinc (ZnCl_2_ 2.0 mM) was titrated into dimeric apo-Zur of (**A**) H36A (40 μM), (**B**) C79SH87A (M-site) mutant (65 μM) or (**C**) H84AE105A (D-site) mutant (53 μM) in the binding buffer (20 mM Tris, pH 7.8, 150 mM NaCl, 0.5 mM TCEP, 3.0 mM EGTA) at 25°C. The upper panel shows representative ITC heat signals generated in the titration as time traces. The raw heat signals were integrated over time and normalized per mol of zinc injected to obtain heats of binding plotted in the bottom panel as a function of molar ratio. The normalized ITC curve was fitted to a single-class site model (continuous line), yielding the binding stoichiometry, affinity, and enthalpy (Supplementary Table S2). In addition, ITC curves were simulated with the binding constants 10-fold higher (dashed line) and lower (dotted line) than the best-fit value.

To examine whether H36 contributes to the binding of zinc to the fourth site, we performed ITC analysis of the interaction of H36A Zur with zinc and compared it with other zinc-binding mutants. The ITC result demonstrates that an H36A apo-Zur dimer binds 4.0 equivalents of zinc, 2.0 equivalents less than the wild type binding (Figure 3A, Figure 1A). The H36A mutation did not change the overall structure of apo-Zur protein, as judged by its circular dichroism spectra being nearly identical to the wild type (Supplementary Figure S5). Therefore, H36 is the critical residue that most likely constitutes the fourth zinc-binding site in Zur, which has not been reported so far.

We measured the zinc binding stoichiometry of two Zur variants with mutations in site 2(M) or site 3(D), two regulatory sites previously identified. The C79SH87A (M-site) and H84AE105A (D-site) mutations caused decrease in zinc binding stoichiometry, leading to 2.6 and 4.0 equivalents of zinc per apo-Zur dimer, respectively (Figure 3B and C). The M-site mutation caused a greater reduction in zinc binding capacity of Zur than the D-site mutation, consistent with its larger effect on the DNA binding activity (24). Regardless of the various binding stoichiometries, the zinc binding affinities are similar within experimental uncertainty among the wild type and mutants except the M-site mutant unexpectedly exhibiting a 3-fold higher affinity (Supplementary Table S2). These results collectively suggest that the effect of the D-site or H36A mutation is localized to eliminate its own zinc binding activity but that the C79SH87A mutation has caused changes in conformation and/or dynamics of the M-site presumably propagating to the nearby D-site and even affecting the H36 site affinity.

A recent structural study (28) suggested a possibility of a salt bridge between a pair of residues near H36 (D37 and H41) at the interface between Zur dimers. Whether H41 can serve as a zinc-binding site was examined by ITC experiment titrating zinc into the H41A mutant. The result yielded the binding isotherm essentially identical to that of the wild type (Supplementary Figure S6), indicating that interdimeric coordination of zinc is unlikely at least in the DNA-unbound state of Zur.

### H36A Zur shows a compromised DNA binding activity

**Figure 4.**
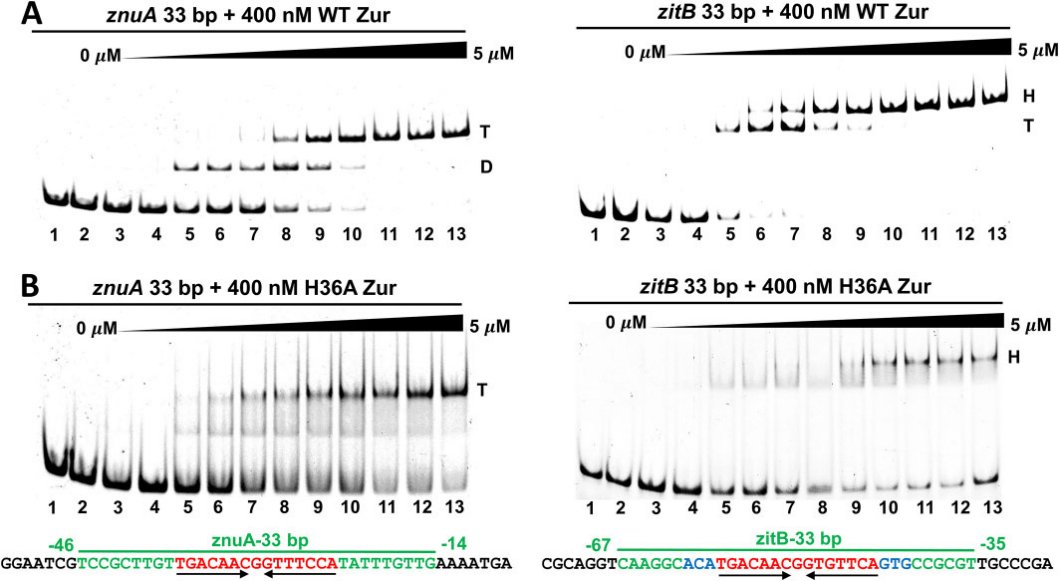
Interaction of WT or H36A Zur with 33 bp *znuA* and *zitB* DNA characterized by EMSA. Either the wild-type (**A**) or H36A (**B**) Zur (400 nM) was incubated with the 33 bp *znuA* (left panels) or *zitB* (right panels) DNA probe (65 nM) labeled with 6-FAM in the presence of varying amounts of ZnSO_4_ (0, 0.1, 0.25, 0.5, 0.75, 1, 2, 2.5, 3, 3.5, 4, 4.5, 5 μM for lanes 2–13). The retarded bands were marked as D (dimer), T (tetramer), or H (hexamer) based on the calculated molecular weights from native PAGE on varying percentage of acrylamide gels (Supplementary Figure S7). DNA bases colored in red and green indicate the 15 bp zurbox motif and its flanking sequences, respectively, in the 33 bp *znuA* or *zitB* DNA probe.

To investigate how the specific zinc-binding to H36 affects the DNA binding activity of Zur, we performed EMSA with the 33 bp DNA probes of *znuA* and *zitB*, each containing one zurbox motif in the middle. At a fixed amount of WT Zur (400 nM) and increasing zinc from 0 to 5 μM, two retarded bands appeared on both the *znuA* and *zitB* DNA probes (Figure 4A). We estimated the molecular size of the retarded bands by native PAGE on varying percentage of polyacrylamide gels (Supplementary Figure S7). The upper band on the *znuA* probe corresponded to a tetrameric Zur-DNA complex and the lower band matched the mobility of a dimeric Zur-DNA complex. On the other hand, the upper band on the *zitB* probe matched the mobility of a hexameric Zur-DNA complex and the lower band corresponded to a tetrameric Zur-DNA complex. It is surprising to observe the formation of the hexameric Zur complex with 33 bp *zitB* DNA by EMSA. This coincides with the cryo-EM observation of a trimer of Zur dimers on 33 bp *zitB* DNA (28). The amount of zinc required to form the intermediate complexes (dimeric for *znuA* and tetrameric for *zitB*) or the final oligomeric complexes appeared similar for the two DNA probes, even though a transition to the final oligomeric complex is observed at a lower zinc concentration on *zitB* than on *znuA* DNA.

The zinc concentrations required for Zur to saturate the 33 bp DNA probes exceed 4.0 equivalents (lane 6) of zinc per Zur dimer (Figure 4A). This suggests that all three regulatory sites, including the H36 site, contribute to achieving the full DNA binding activity of Zur. Accordingly, we observed the weakened binding activities of the H36A mutant for both the *znuA* and *zitB* DNA probes, as demonstrated in the EMSA profile of faint smeared complex bands throughout the zinc concentration range examined (Figure 4B). Because a higher zinc concentration was required for H36A Zur to form the hexameric complex on *zitB* DNA than to form the tetrameric complex on *znuA* DNA, the H36A mutation appears to affect the oligomeric binding on *zitB* DNA to a larger extent.

### Effect of the zurbox-flanking sequences on the oligomeric binding of Zur

**Figure 5.**
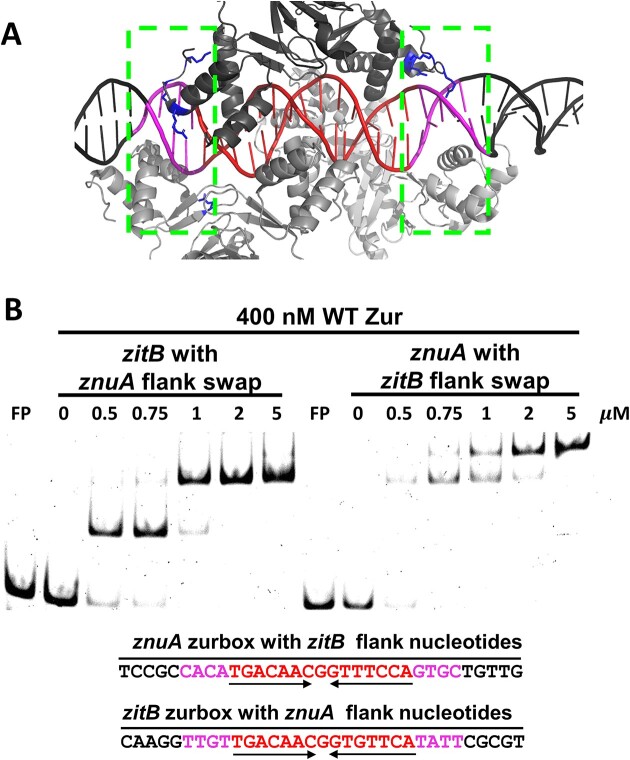
Effects of the sequences flanking the zurbox on the DNA binding mode of Zur. (**A**) The cryo-EM structure of the complex between hexameric *Sc*Zur and 33 bp *zitB* DNA (PDB code 7vo0) (25) showing cationic side chains of K and R (blue sticks) within 6 Å of DNA phosphates in the flank region (colored magenta) of the *zitB* zurbox (colored red). (**B**) EMSA analysis of binding of WT Zur to 33 bp *zitB* DNA with the *znuA* flanking sequences (4 nt each) or to *znuA* DNA with *zitB* flanking sequences (4 nt each). 400 nM WT Zur was treated with varying concentrations of zinc (0, 0.5, 0.75, 1, 2, 5 μM) in the presence of 65 nM 6-FAM labelled DNA fragments. The retarded bands were marked as D (dimer), T (tetramer), and H (hexamer) based on the calculated molecular weights from native PAGE with different acrylamide percentages. DNA bases colored in red and magenta indicate the 15 bp Zur-box motif and the swapped DNA bases, respectively.

Even though the zurbox 7–1–7 sequence differs only by two nucleotides between *znuA* and *zitB*, the oligomeric binding of Zur dimer turns out to be very different. It is notable that the sequences flanking the zurbox are quite different between *znuA* and *zitB*. The immediate flanking sequences of *znuA* are highly AT-rich, whereas *zitB* has less AT-rich sequences (Figure 4A and B). A recent cryo-EM structure of the Zur-*zitB* DNA complex shows significant interactions between Zur and the flank regions (Figure 5A). The solvent accessible surface area buried in these regions account for about 20% (1837 Å^2^) of the total binding interface. Likewise, seven cationic side chains of Zur are within 6 Å of DNA phosphates in the flank regions for potential electrostatic interactions. Thus, to test the effect of the flanking sequences on the DNA binding property of Zur, we swapped the immediate 4 flanking nucleotides of the zurbox between *znuA* and *zitB*, and examined binding of Zur to these chimeric DNA probes by EMSA (Figure 5B). Remarkably, the oligomeric state of Zur at saturation was switched between the two DNA probes. Therefore, the proximal 4 nucleotides flanking the 7–1–7 zurbox determines the binding stoichiometry of the interaction between Zur and *zitB* or *znuA* DNA.

### Determination of the DNA binding affinity of WT and H36A zur by ITC

**Figure 6.**
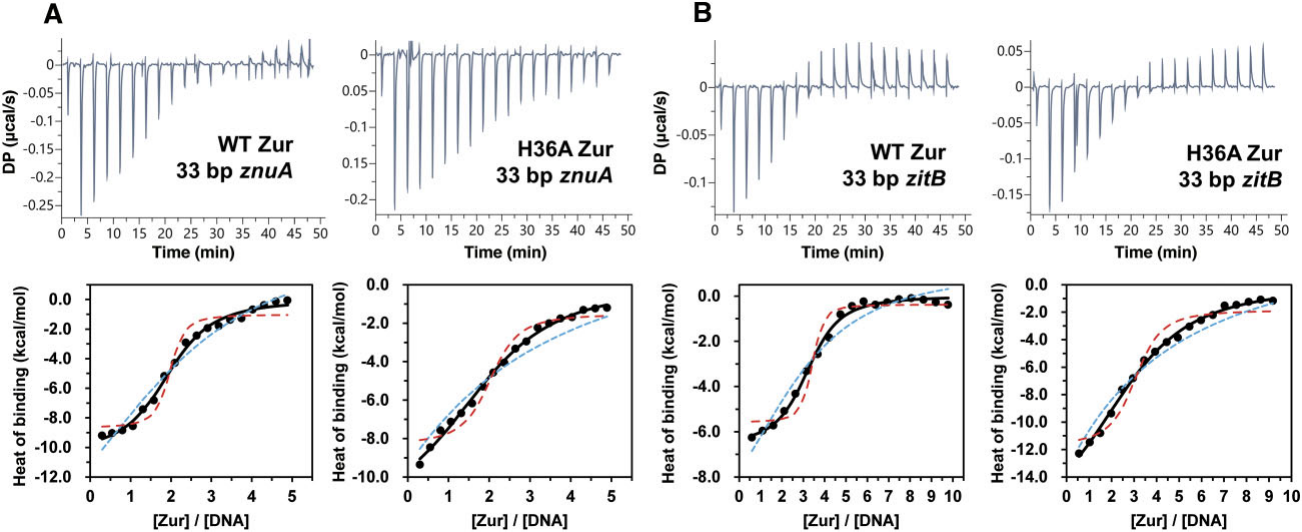
Determination of the DNA binding affinity of wild type and H36A Zur by ITC. Wild type or H36A Zur containing 4.0 equivalents of zinc per dimer was titrated into 33 bp *znuA* (**A**) or *zitB* (**B**) DNA in the binding buffer containing 20 mM HEPES (pH 7.8), 150 mM NaCl and 0.5 mM TCEP at 25°C. The raw heat signals were integrated over time and normalized per mol of zinc injected to obtain heats of binding plotted in the bottom panel as a function of molar ratio. The normalized ITC curve was fitted to a single-class site model (continuous line), yielding the binding stoichiometry, affinity, and enthalpy (Supplementary Table S3). In addition, ITC curves were simulated with the binding constants 10-fold higher (red dashed line) and lower (blue dotted line) than the best-fit value. For direct comparison between WT and H36 Zur, superposition of their ITC curves for each promoter is provided in Supplementary Figure S8.

The apparent DNA binding affinity of zinc-bound WT or H36A Zur was quantified by ITC (Figure 6 and Supplementary Figure S8). WT or H36A Zur was titrated into 33 bp *znuA* DNA in the presence of 4.0 equivalents of zinc per dimer. Inclusion of a higher amount of zinc caused precipitation of Zur, precluding reliable measurement. The analysis of the ITC curves by a single-class site model demonstrated that both WT and H36A Zur bound the *znuA* DNA with a 2:1 (Zur dimer:DNA) stoichiometry. The best-fit DNA binding affinity was higher for the wild type (8.7 (± 1.2) × 10^5^ M^−1^) than for H36A Zur (3.3 (± 1.0) × 10^5^ M^−1^) (Figure 6A and Supplementary Table S3). We then examined the interaction between Zur and 33 bp *zitB* DNA. The analysis of the ITC data demonstrated that both WT and H36A Zur bound 33 bp *zitB* DNA with a 3:1 stoichiometry (Figure 6B). The hexameric (trimer of dimers) binding of Zur to *zitB* DNA coincides with the previous observation made by cryo-EM (28). However, the affinity of H36A Zur for the *zitB* probe (2.6 (± 0.7) × 10^5^ M^−1^) was 7-fold lower than that of WT Zur (1.8 (± 0.2) × 10^6^ M^−1^) (Figure 6B and Supplementary Table S3). The effect of H36A mutation on the affinities quantified by ITC are consistent with our EMSA results showing that the H36A mutation deteriorates the binding activities of Zur for both *znuA* and *zitB* DNA with a larger effect on *zitB*.

### Thermodynamic model for the interaction among Zur, zinc, and DNA demonstrating a high affinity of zinc-saturated Zur for DNA

The binding stoichiometries of Zur determined by ITC for *znuA* (2:1) and *zitB* (3:1) DNA agree with the tetrameric and hexameric binding of Zur to these DNA probes observed in EMSA. However, the ITC experiments were performed in the presence of 4.0 equivalents of zinc ions per Zur dimer insufficient to occupy all three regulatory sites of Zur. At similar zinc concentrations, the formation of tetrameric or hexameric Zur on *znuA* or *zitB* DNA, respectively, was insignificant in EMSA. This apparent discrepancy may arise from the lower concentrations of DNA (65 nM) and Zur (200 nM in dimer) used in EMSA, presumably driving dissociation of the higher-order assemblies with micromolar affinity (Supplementary Table S3). Nonetheless, even at such low protein and DNA concentrations, increasing zinc concentration drove the formation of the tetrameric or hexameric Zur-DNA complexes (Figure 4A). Thus, the DNA binding affinity of Zur with all three regulatory sites occupied by zinc must be greater than those of the partially occupied Zur species.

**Figure 7.**
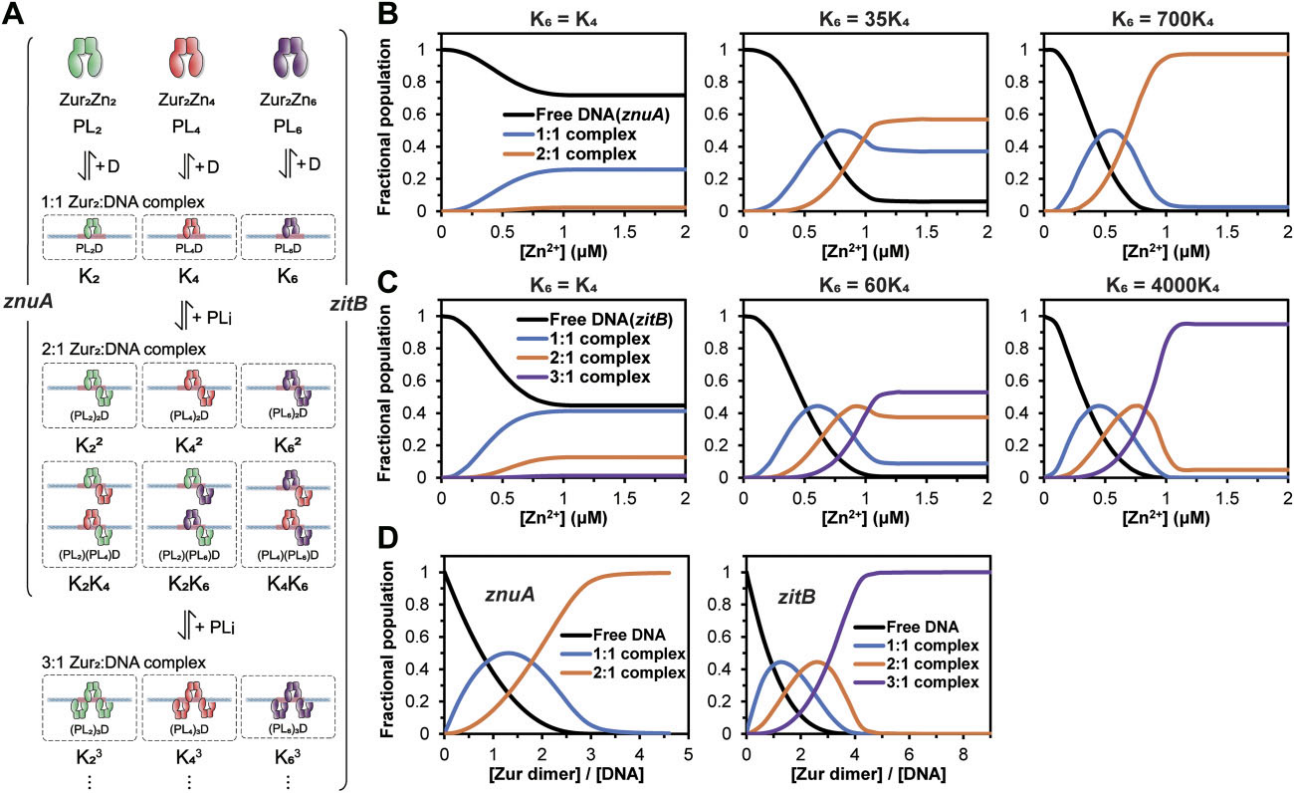
Thermodynamic model for coupling between the zinc binding and DNA binding equilibria of Zur. (**A**) Schematic illustration of the thermodynamic model. Dimeric Zur, DNA with a consensus sequence, and zinc are denoted by *P*, *D* and *L*, respectively. A dimer contains three pairs of regulatory zinc binding sites. The number of regulatory zinc ions bound per dimer are designated the subscript *i* and they are randomly distributed over the six binding sites. Although *PL*_1_, *PL*_3_ and *PL*_5_ are not shown in the illustration for simplicity, these states were included in our model as described in Supplementary Information S2. Zur of each zinc-bound state binds the consensus zurbox with a specific binding affinity *K_i_*. Dimeric Zur can form 1:1 to 2:1 or 3:1 complex with *znuA* or *zitB* DNA, respectively. In the 1:1 complex, although only a single DNA-bound configuration is shown for a Zur dimer with a given number of zinc bound, the dimer can in principle sample two or three potential binding sites on *znuA* or *zitB* DNA, respectively. Furthermore, the *n*:1 complex is an ensemble of multiple states with various combinations of *PL*_i_ bound to DNA as shown in the illustration for the 2:1 complex (A full set of molecular states of the 3:1 complex is illustrated in Supplementary Figure S11A). Such statistical features are explicitly considered in our model (Supplementary Information S2). (**B** and **C**) Population distribution among free DNA (B: *znuA*; C: *zitB*), 1:1, 2:1, and 3:1 complexes as a function of zinc concentration. *K_6_* was varied from *K_4_* to a value at which the 2:1 (for *znuA*) or 3:1 (for *zitB*) complex becomes predominant (95% of total DNA population) at high zinc concentrations. How the *K_i_* values were assigned in the simulation is described in detail in Supplementary Information S2. (**D**) Population distribution among free DNA, 1:1, 2:1 and 3:1 complexes as a function of molar ratio [Zur dimer]/[DNA] at a fixed zinc concentration.

In order to demonstrate such critical insights and predict the DNA binding affinity of zinc-saturated Zur, we constructed a thermodynamic model for coupling between the zinc binding and DNA binding equilibria of Zur (Figure 7). In this model, the Zur population comprises free and DNA bound forms of Zur dimer with the binding stoichiometries determined by EMSA and ITC (Figure 7A). In turn, each form is an ensemble of the microstates with various numbers and distributions of zinc ions bound over the three pairs of regulatory sites in dimeric Zur (see Supplementary Information S2 for a detailed description of the model). Then, for given concentrations of dimeric Zur (*P*), DNA (*D*) and zinc (*L*), the population distribution among these molecular states can be calculated from the zinc binding constant (Supplementary Table S2) and DNA binding constants (*K*_*i*_) of *PL*_i_. The DNA binding constant quantified by ITC in the presence of 4.0 equivalents of zinc per Zur dimer (Supplementary Table S3) was assigned to the DNA binding affinity (*K*_4_) of *PL*_4_. Since it is unlikely that, on the basis of the previous and current mutation studies (21), *PL*_2_ is active in interaction with DNA, subtle binding affinities (≤10^4^ M^−1^) were assigned to this state. Finally, we examined a wide range of DNA binding affinity for dimeric Zur with three regulatory sites occupied by zinc (*PL*_6_).

Using these interaction parameters and the concentrations of Zur and DNA used in EMSA, we calculated the fractional populations of the free and Zur-bound DNA molecules as functions of zinc concentration (Figure 7B and C). Assigning the equivalent affinity to *PL*_4_ and *PL*_6_ resulted in overall low DNA binding activity and no significant oligomeric binding of Zur on both DNA probes throughout the zinc concentration range used in EMSA (First plots in Figure 7B and C). Instead, it was necessary to significantly increase the DNA binding affinity of *PL*_6_ to form oligomeric Zur-DNA complexes at high zinc concentrations (Figure 7B and C). Notably, an affinity of *PL*_6_ about three orders of magnitude higher than *PL*_4_ drives transition from dimeric to tetrameric binding on *znuA*, or from tetrameric to hexameric binding on *zitB*, recapitulating the qualitative features of the EMSA results (*K*_6_ = 700*K*_4_ and 4000*K*_4_ in Figure 7B and C, respectively). In addition, we performed simulations parallel to the ITC experiments where Zur is titrated into DNA (3 μM) in the presence of 4.0 equivalents of zinc per Zur dimer (Figure 7D). Even with this fixed amount of zinc insufficient to saturate Zur, the micromolar concentrations of Zur and DNA enabled oligomeric binding at high molar ratio of Zur to DNA (Figure 7D), consistent with the experimental data.

To validate the high DNA binding affinity predicted for zinc-saturated Zur, we performed EMSA by incubating increasing amount of Zur with the fluorescently labeled *zitB* DNA probe in the presence of the excess amount of zinc (>6.0 eq. per Zur dimer) (Supplementary Figure S9A). Much lower concentrations of Zur were used in the EMSA experiment than in ITC, avoiding protein aggregation. Fractional distribution among free, tetrameric (dimeric Zur:DNA = 2:1), and hexameric Zur-bound DNA (dimeric Zur:DNA = 3:1) was quantified from the band intensities and analyzed to estimate the DNA binding constants for formation of the 2:1 and 3:1 complexes (Supplementary Figure S9B). This analysis yielded low to sub-nanomolar affinities much tighter than that of partially saturated Zur (Figure 6B) but within the same order of magnitude as the model-predicted value of zinc-saturated Zur. Therefore, our thermodynamic model corroborates all EMSA and ITC results, demonstrating the strongest DNA binding activity of Zur with all three regulatory sites occupied by zinc. Moreover, the simulations and experimental data collectively show that the formation of the oligomeric Zur-DNA complex is intricately regulated by concentrations of Zur, DNA and zinc, which may be relevant to the transcriptional regulation *in vivo* (see Discussion).

### H36A Zur does not form extended multimeric binding in the upstream region of *zitB*

**Figure 8.**
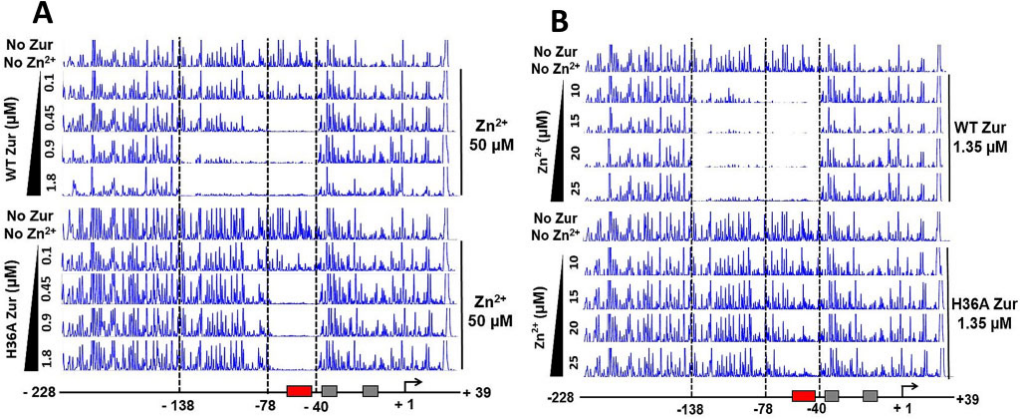
Contribution of the H36 site to the extended binding of Zur to the upstream of the *zitB* zurbox examined by DNase I footprinting. The 267 bp *zitB* DNA probe (−228 to + 39 nt relative to the transcription start site (TSS)) was incubated with varying amounts (0.1, 0.45, 0.9, 1.8 μM) of wild-type (upper panel) or H36A (lower panel) Zur in the presence of 50 μM ZnSO_4_ (**A**), or with varying amounts (10, 15, 20, 25 μM) of ZnSO_4_ in the presence of 1.35 μM Zur (**B**). The protection pattern was monitored by capillary electrophoresis. The protected region (−40 to −138 nt relative to TSS) is marked with dotted lines. The control DNase I footprinting pattern is presented for the *zitB* DNA probe without Zur and added zinc.

The full activation of the *zitB* gene is achieved by binding of multiple copies of Zur to the upstream of the zurbox at high zinc concentrations (21). To test whether the zinc binding to the H36 site is required for the upstream DNA binding of Zur, we performed DNase I footprinting experiments of a 267 bp *zitB* DNA probe (−228 to + 39 nt from the transcription start site) with WT and H36A Zur. The protection patterns were visualized by capillary electrophoresis and fluorescence scanning. Either increasing amount of Zur protein (0.1–1.8 μM) was incubated with the *zitB* DNA probe at 50 μM of ZnSO_4_ (Figure 8A), or increasing amount of ZnSO_4_ (10–25 μM) was added to the binding mixture containing Zur at 1.35 μM (Figure 8B). WT Zur initially protected a region between −78 and −40 nt position containing the zurbox motif. With increasing protein or zinc concentration, the protection was further extended toward the upstream up to −138 nt position (Figure 8A and B), consistent with the previous observation (21). On the other hand, we observed that H36A Zur protected only the promoter-proximal region (−78 to −40), with a lower efficiency as compared with the wild type (Figure 8B). Even at the highest concentrations of Zur (1.8 μM) and zinc (50 μM), no extended multimeric binding of Zur to the distal upstream region of the *zitB* promoter was observed for H36A Zur. Therefore, H36 is critical for the zinc dependent binding of Zur to both the consensus zurbox and its upstream region.

## Discussion

In this study, we discovered the fourth zinc-binding site in *S. coelicolor* Zur (*Sc*Zur) that serves a regulatory function. Titration of zinc into apo-Zur with two prebound structural zinc ions revealed that dimeric Zur binds 6.0 equivalents of zinc (Figure 1A) with a binding affinity in the femtomolar range per site. This affinity range is comparable to those observed in other bacterial Zur, being in the sub-picomolar range (13), and tighter than those of other zinc-responsive metalloregulators, such as ZntR, CzrA and AdcR, that are in the nano- to picomolar range (32–34). In our experimental conditions, the ITC binding isotherms for the zinc-Zur interaction are apparently monophasic, suggesting similar affinities or moderate heterogeneity (<20-fold difference in affinity) among the three regulatory sites. This contrasts with a clear biphasic ITC curve observed for the interaction between *S. aureus* CzrA and zinc (32), the analysis of which revealed negative cooperativity for sequential binding of zinc ions to a pair of regulatory sites present in a CzrA homodimer. Zur and other zinc-responsive bacterial proteins exhibited no or negative cooperativity among regulatory sites differing in zinc binding affinity by 1–2 orders of magnitude (13,25,34,35). Understanding molecular basis for such a various range of cooperativity (from none to 100-fold negative coupling) measured for different zinc binding proteins requires further structural and thermodynamic investigations at high resolution.

Zur proteins reported so far have one structural site (C-site or site 1) and one to two regulatory sites per monomer (13). Each zinc-binding site tetracoordinates zinc ions. A structural site is coordinated by four cysteines and regulatory sites have an N_2_OS composition (23,24,27,36). Through mutational study, we showed that histidine-36 is a critical residue constituting the fourth zinc-binding site in Zur (Figure 3A). This residue is well conserved among Zur of all actinobacteria examined, along with the universally conserved residues in the first and third helices functioning in DNA recognition (Supplementary Figure S10). A single surface-exposed aspartate, glutamate, or histidine residue is capable of binding a zinc ion (37,38). Moreover, the crystals grown in the presence of zinc ions have revealed zinc ions bound on the protein surface (39). Considering that H36 is exposed to solvent at the DBD of Zur, it is conceivable that H36 alone, or together with water molecules and yet unidentified residues, can interact with a zinc ion. It is perplexing that zinc bound to this site was not observed in the previous structural studies (24,28). Provided that the site is solvent-accessible and coordinated by fewer residues than other regulatory sites, binding of zinc at this site is likely dynamic and prone to perturbations by any changes in solution condition including temperature and cosolute concentration. Hence, zinc ions may have been dissociated from the H36 site under the conditions for crystallization or preparation of EM grids.

Our EMSA and ITC results showed a compromised binding activity of H36A Zur for 33 bp *znuA* and *zitB* DNA, demonstrating that the binding of zinc to H36 is critical for interactions of Zur with the specific DNA probes. (Figure 4 and 6). Likewise, our previous study has reported M and D-sites as important in the Zur-DNA interactions (24). Therefore, all three regulatory zinc binding sites contribute to the full DNA binding activity of Zur, consistent with the high zinc concentrations required in EMSA for Zur to saturate the 33 bp DNA probes (Figure 4). We integrated all these results to construct a thermodynamic model for quantitative illustration of coupling between the zinc binding and DNA binding equilibria of Zur (Figure 7). This model predicted a remarkable enhancement in specific DNA binding affinity of Zur upon occupation of all three regulatory sites by zinc. About a 1000-fold increase in DNA binding affinity from Zur_2_Zn_4_ to Zur_2_Zn_6_ drove the tetrameric or hexameric binding of Zur to *znuA* or *zitB* DNA, respectively. It is noteworthy that independent Zur titration experiments by EMSA (Supplementary Figure S9) substantiated our thermodynamic model, yielding a DNA binding affinity of zinc-saturated Zur within the same order of magnitude as the model-predicted value of nanomolar range.

A sequential increase in DNA binding affinity of Zur with increasing zinc occupancy may be a molecular underpinning of the graded response of Zur previously reported (24). For some promoters like *rpmG2* or SCO7682, DNA binding by unsaturated Zur may allow their repression. On the other hand, for promoters like *zitB* or *znuA*, the DNA binding by fully zinc-saturated Zur may be needed for their full activation or repression, respectively. Although our current model and data do not specify the order of the three regulatory sites in zinc binding, different zinc binding affinities among them could also contribute to expanding the sensory range, resulting in graded response. If the potential heterogeneity of up to 20-fold difference in zinc binding constant exists as our fitting analysis suggests, the M-site is likely to bind zinc first with the highest affinity to prime Zur toward a closed conformation active in DNA binding as proposed before (24).

The strong coupling between the zinc binding and DNA binding equilibria may arise from allosteric conformational changes in DBDs of Zur yet to be discovered. Even though our thermodynamic model does not explicitly invoke cooperativity between Zur dimers bound on DNA, such potential protein-protein interactions may have been embedded, together with an intrinsic affinity for DNA, in the binding constant *K*_i_. Then, the coupling (*K*_6_/*K*_4_) may involve stabilization of protein-protein interactions on DNA by saturation of Zur with zinc. Of note, the H36 zinc binding site is adjacent to the residues (D37 and H41) mediating cooperativity between DNA-bound Zur dimers through the salt bridge formation observed in structural studies (27,28).

**Figure 9.**
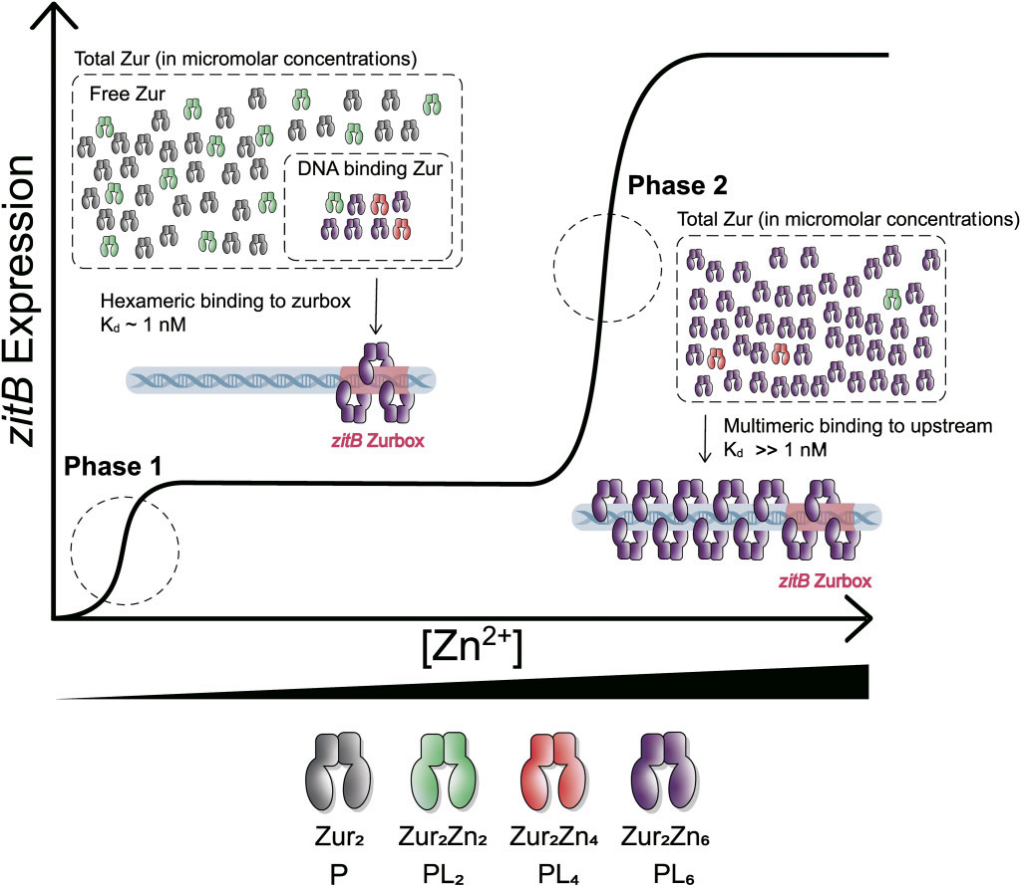
Molecular basis for the zinc-dependent biphasic activation of the *zitB* gene by Zur *in vivo*. A thermodynamic description for each phase of the *zitB* gene activation is shown on a schematic zinc-dependent gene expression profile. In phase 1, while the majority of Zur exists as apo- or low zinc-occupancy states, a small fraction of Zur interacts with the zurbox (as a trimer of dimer) and transitions to a high zinc-occupancy state due to its strong affinity for the zurbox. In phase 2, a further increase in zinc concentration fully saturates the entire population of Zur, culminating in binding of multiple copies of Zur to the upstream DNA containing multiple low-affinity sites. A simulation qualitatively recapitulating such a sequential activation of the *zitB* gene is presented in Supplementary Figure S12.

The *zitB* gene responds to a wide range of concentration changes of zinc added to the growth media. While the *zitB* gene is partially activated under a low (sub-femtomolar in the presence of chelator TPEN) zinc condition (phase 1), increasing zinc from 10 to the hundred micromolar concentration further elevated the gene expression level by 6- to 7-fold (phase 2) (21). Such an *in vivo* biphasic activation of the *zitB* gene is mediated by sequential binding of the zurbox and its upstream region by Zur with increasing zinc concentration as observed in our footprinting experiments (Figure 8A and B). Our thermodynamic model provides a potential molecular mechanism for the biphasic or sequential activation of the *zitB* gene by Zur (Figure 9). Initially, at zinc concentrations far below a saturating level, the majority of Zur would be free of or partially occupied by zinc (Phase 1 in Figure 9). However, in a population of Zur interacting with the zurbox, the zinc binding equilibrium would substantially shift toward a high-occupancy state because of its strong affinity for the zurbox. This population is very small but sufficient in amount to saturate the zurbox since the total Zur concentration (∼4 μM in *S. coelicolor* cell) is much greater than that of DNA (21). During the saturation of the zurbox, the upstream DNA, which contains multiple nonspecific (or lower-affinity) binding sites for Zur (40), would be mostly free and unprotected because their affinity is much lower than that of the zurbox. However, as the zinc concentration is further increased, the entire population of Zur would be saturated with zinc (Phase 2 in Figure 9). Then, the concentration of active Zur becomes high enough to effectively bind the multiple low-affinity sites in the upstream region (see Supplementary Figure S12 for a coarse-grained thermodynamic simulation of the sequential protection of the zurbox and its upstream region). Therefore, the physiological concentrations of Zur and DNA and their binding affinities appear poised for the thermodynamic coupling to drive the zinc-dependent biphasic activation of the *zitB* gene. Such a physiological impact of the interplay among thermodynamic variables has been recently demonstrated in predicting the gene expression level as a function of competition between specific and nonspecific sites for given concentrations of transcription factors (41).

Response to a wide range of zinc concentration change by Zur acting both as a repressor and an activator has also been reported in *Synechococcus*, sp. WH8102 recently (22). As zinc availability increases, SynZur starts to repress *znuABC* at low (picomolar range) zinc, and activates *bmtA* gene encoding metallothionein for zinc storage to a full level at micromolar zinc. In this context, activation mechanism of a TetR-family metalloregulator SczA, which activates a zinc export gene (*sczD*) in excess zinc and represses the same gene at low zinc, will be interesting to investigate (42).

Our results clearly demonstrated the effect of the sequences flanking zurbox on the DNA binding stoichiometry of Zur. Swapping flanking regions of the zurbox between *znuA* and *zitB* DNA revealed that 4 nucleotides immediately flanking the zurbox are responsible for the final oligomeric binding pattern of Zur (Figure 5). The effect of the flanking regions on the interaction between protein and DNA has been reported for the transcriptional regulators Cbf1 and Tye7 in *S. cerevisiae* (43). The most prominent difference in the flanking sequences of the zurbox between *znuA* and *zitB* is the abundance of A/T (CATT, TATT) in *znuA*. Generally AA, TT, and AT sequences are known to induce a narrow minor groove and negative roll angle (44–46), causing the helical axis of the duplex DNA to bend (45). In the crystal structure of EcZur-*znuA* complex, EcZur binding caused a bend at bases 15 (18.3 Å) and 16 (16.8 Å), which are larger than 11.4 Å for B-form DNA (27). On the other hand, *Sc*Zur caused only a slight bend in the *zitB* DNA compared with the EcZur-*znuA* complex (28). This suggests that the T-rich flanking sequence of the *znuA* zurbox may induce a distinct DNA structure, such as narrower minor groove and bending, which prevents hexameric Zur binding.

In conclusion, our study discovered a novel zinc-binding site in *S. coelicolor* Zur containing a key histidine residue (H36). This zinc-binding site, together with other known regulatory metal sites, is coupled to the DNA binding activity of Zur. A thermodynamic model was developed for quantitative description of such coupling, further rationalizing the transcriptional regulation of the *zitB* gene *in vivo* and the graded response of distinct zinc-responsive genes. The mode of action of *Sc*Zur in adapting to a wide range of zinc concentration change as a repressor and an activator provides new insights in understanding the behavior of ligand-sensing proteins in general. Finally, in parallel to a recent cryoEM study (28), further investigations are required to elucidate a structural mechanism for interaction between upstream-bound oligomeric Zur and RNA polymerase driving the second phase activation of the *zitB* gene.

## Supplementary Material

gkae079_Supplemental_File

## Data Availability

The mathematical equations required for the simulation of the thermodynamic model are provided with detailed derivations in Supplementary Information S2. The binding assay data analyzed in this study are available upon request.
