# Supplementary material for: Activation of zinc uptake regulator by zinc binding to three regulatory sites
Source: Nucleic Acids Res. 2024 Feb 13;52(8):4185–97. doi: 10.1093/nar/gkae079 (PMC11077047; doi:10.1093/nar/gkae079)
Supplement: gkae079_Supplemental_File [file gkae079_supplemental_file.pdf]

Supplementary Information and Data

Activation of zinc uptake regulator by zinc binding to three regulatory sites

Yunchan Choi[1], Junseock Koh[2]*, Sun-Shin Cha[3], and Jung-Hye Roe[1]*

[1]*Laboratory of Molecular Microbiology, and* [2]*Laboratory of Biophysical Chemistry, School of Biological Sciences, College of Natural Science, Seoul National University, Seoul 151-742,* [3]*Protein Research Laboratory, Department of Chemistry and Nanoscience, Ewha Womans University, Seoul 03760, Republic of Korea*

*To whom correspondence should be addressed. Tel: +82 2 880 6706; Fax: +82 2 882 6706; Email: jhroe@snu.ac.kr

*Correspondence may also be addressed to Junseock Koh. Tel: +82 2 880 4425; Email: junseockkoh@snu.ac.kr

Present Address: Yunchan Choi, School of Biological Sciences, Seoul National University College of Natural Science, Seoul 08826, Korea

Keywords: zinc homeostasis, repressor and activator, zinc export, regulatory zinc binding, oligomerization, thermodynamic coupling

Supplementary Information S1

Correction of the zinc binding affinity of Zur for the competition effect from EGTA

The experimental conditions used for determination of the zinc binding affinity of Zur include EGTA to prevent adventitious zinc binding as well as to bring the apparent affinity within a range measurable in ITC. We used two independent methods to correct the binding parameters quantified in ITC experiments for the competition between Zur and EGTA for zinc binding, and obtained the intrinsic binding affinities and enthalpies consistent between these two methods (Supplementary Table S2 and S4). We denote a zinc binding site of Zur, zinc, and EGTA by *P*, *L*, and *C*, respectively, in this section for simplicity.

In a conventional method, namely *post hoc* analysis (1,2), the binding constant quantified in ITC is defined as

$$K_{ITC} = \frac{[PL]}{[P][L']} \qquad \text{(Eq. S1)}$$

where $[L']$ is the concentration of zinc unbound to Zur, including free (*L*) and competitor-bound zinc (*CL*) with an affinity of K_c.

$$[L'] = [L] + [CL] = [L] + K_c[L][C] \qquad \text{(Eq. S2)}$$

Then, K_{ITC} is given by

$$K_{ITC} = \frac{[PL]}{[P][L]}\frac{[L]}{[L']} = K\frac{1}{1 + K_c[C]} \qquad \text{(Eq. S3)}$$

where *K* is the intrinsic zinc binding affinity of Zur. The value of K_C was calculated from an equation previously derived (3) by taking into account the NIST approved zinc binding affinity and protonation of EGTA base (K_C = 8.7x10^9 M^{-1} at pH 7.8). The free EGTA concentration [*C*] can be approximated as $[C]_{total}$ (3 mM) because $[C]_{total} >> [L]_{total}$ in the ITC reaction cell.

Differentiating K_{ITC} with respect to temperature yields the binding enthalpy quantified in ITC (assuming that [*C*] is $[C]_{total}$ and independent of temperature)

$$\Delta H^{\circ}_{ITC} = -R\frac{\partial lnK_{ITC}}{\partial(1/T)} = \Delta H^{\circ} - \Delta H^{\circ}_{C}\frac{K_c[C]}{1 + K_c[C]} \qquad \text{(Eq. S4)}$$

However, because $K_C[C] >> 1$,

$$\Delta H^{\circ}_{ITC} = \Delta H^{\circ} - \Delta H^{\circ}_{C} \tag{Eq. S5}$$

ΔH°_{C} was obtained directly from the titration of zinc into EGTA (Supplementary Figure S13).

In the second approach, we incorporated the competition equilibrium into the model used in fitting the ITC data to directly quantify the intrinsic binding affinity and enthalpy. The ITC signal measured at the *i*th injection ($q_{p,i}$) comprises the heat arising from the *PL* formation ($q_{p,i,PL}$) and from the *CL* dissociation ($q_{p,i} = q_{p,i,PL} + q_{p,i,CL}$). These two contributions are given by the following standard heat equation (4).

$$q_{p,i,X} = \frac{\Delta H^{o}_{X} V_{cell}}{[L]_{syr} V_{inj}} \{[X]_i - [X]_{i-1} + \frac{V_{inj}}{V_{cell}}\left(\frac{[X]_i + [X]_{i-1}}{2}\right)\} \tag{Eq. S6}$$

In Eq. S6, $\Delta H_X°$ is the standard enthalpy change for formation (or dissociation) of complex *X*; V_{cell} is the volume of the reaction cell; $[L]_{syr}$ is the molar concentration of zinc in the titration syringe; V_{inj} is the injection volume; $[X]_i$ and $[X]_{i-1}$ are the equilibrium molar concentrations of the complex formed (or complex dissociated) in the reaction cell after *i*th and *i-1*th injections, respectively. These concentrations are obtained by solving the following mass balance equation of *L* for the concentration of free *L* with given values of *K*, K_c, and *n* (= number of zinc binding sites per Zur).

$$[L]_{total} = [L] + [PL] + [CL] \tag{Eq. S7a}$$

$$[L]_{total} = [L] + \frac{nK[L]}{1 + K[L]}[P]_{total} + \frac{K_c[L]}{1 + K_c[L]}[C]_{total} \tag{Eq. S7b}$$

[*CL*] obtained with the solved value of [*L*] is the concentration of *CL* formed in the ITC reaction cell. The concentration of dissociated *CL* ($[CL]_{disso}$) is the difference between [*CL*] and the concentration of *CL* preformed in the titration syringe and then diluted into the reaction cell ($[CL]_{inj}$).

$$[CL]_{disso} = [CL] - [CL]_{inj} \tag{Eq. S8}$$

In turn, $[CL]_{inj}$ after the *i*th injection is calculated by the following equations.

$$[CL]_{inj} = [CL]_s \frac{iV_{inj}}{V_{cell}}(1 - \frac{iV_{inj}}{2V_{cell}}) \quad \text{(Eq. S9a)}$$

$$[CL]_s = \frac{([C]_{total,s} + [L]_{total,s} + 1/K_c - \sqrt{([C]_{total,s} + [L]_{total,s} + 1/K_c)^2 - 4[L]_{total,s}[C]_{total,s}}}{2} \quad \text{(Eq. S9b)}$$

The bracket terms with the subscript *s* (e.g. $[C]_{total,s}$) are the concentrations in the titration syringe. Finally, a set of the thermodynamic parameters K, K_c, n, $\Delta H_{PL}°$, and $\Delta H_{CL}°$ yielding $q_{p,i}$ which best fits the observed binding heat was obtained by the nonlinear least-squares minimization method in Igor Pro 8.02 (WaveMatrics) utilizing the Levenberg-Marquadt algorithm.

Deprotonation of metal-coordinating amino acids is often coupled to protein-metal interactions. In such cases, the [*P*] term in the denominator of Eq. S1 includes the concentrations of all (de)protonated states of *P* (i.e., proton binding polynomial).

$$\begin{aligned}[P] &= [P_{deprot}] + [PH] + [PH_2] + \cdots \\ &= [P_{deprot}](1 + 10^{pK_{a1}-pH} + 10^{pK_{a1}+pK_{a2}-2pH} + \cdots)\end{aligned} \quad \text{(Eq. S10)}$$

Therefore, the apparent metal-binding affinity of a protein is a function of pH and deprotonation constants of metal-coordinating amino acids (pK_{ai}). So is the binding enthalpy with an additional contribution from the ionization heat of a buffer (see Supplementary Figure S2 for correction of this additional contribution). Calculation of pH/buffer-independent metal binding thermodynamics requires extensive protein-metal titration data at various pH and buffers (5). While the buffer ionization heat has been corrected in several protein-metal binding studies (2,6,7), the corresponding correction of the binding affinity is exceedingly rare (Exemplary cases can be found in the studies of the interaction between single strand DNA binding protein (SSB) and DNA (8)). Such a meticulous correction goes beyond the scope of our study. Instead, we specify the standard condition (Tris pH 7.8, 25 °C) for our measurement. Moreover, using the measured zinc binding affinity (with the EGTA competition corrected) in the subsequent modeling does not affect our overall qualitative conclusion regarding coupling between the zinc binding and DNA binding equilibria of Zur.

Supplementary Information S2

Thermodynamic model for coupling between the zinc binding and DNA binding equilibria of Zur

In our thermodynamic model for coupled zinc (*L*) and DNA (*D*) binding of dimeric Zur (*P*), the population of Zur comprises free form, zinc-bound forms, and DNA-bound forms (Figure 7A). Among zinc-bound forms, PL_1, PL_3, and PL_5 were included in our modeling even though they were not explicitly shown in the schematic illustration (Figure 7A). In addition, because the zinc binding sites appear to be indistinguishable from one another in terms of affinity, each zinc-bound form (PL_i) is an ensemble of dimeric Zur with *i* zinc ions bound randomly over the three pairs of regulatory sites (a total of six binding sites). Then, the zinc binding equilibrium is completely described by the binding polynomial Z_{PL} which is the sum of the statistical weights or probabilities of free and zinc-bound forms.

$$Z_{PL} = (1 + K[L])^6 \qquad \text{(Eq. S11)}$$

In this equation, *K* is the intrinsic zinc-binding affinity of Zur determined by ITC (Supplementary Table S2). The concentration of each zinc-bound form is given by

$$[PL_i] = {}_6C_i(K[L])^i[P] \qquad \text{(Eq. S12)}$$

Likewise, the equilibrium for the interaction of zinc-bound Zur with DNA is defined by the binding polynomial Z_{PD} which is the sum of the statistical weights of free and Zur-bound DNA.

$$Z_{PD} = \left(1 + \sum_{i=1}^{6} K_i[PL_i]\right)^N \qquad \text{(Eq. S13)}$$

In Eq. S13, *N* is the DNA binding stoichiometry of Zur dimer (*N*=2 and 3 for *znuA* and *zitB* DNA, respectively) and K_i is the DNA binding constant of PL_i. As illustrated in Figure 7 and Supplementary Figure S11, for a given number of Zur dimers bound per DNA, the Zur-DNA complex is an ensemble of multiple states with various combinations of PL_i bound to DNA. The terms in an expanded form of Eq. S13 correspond to the statistical weights of these states. The concentrations of all molecular states present in the three-

component system can be calculated by solving the following mass balance equations of P and L for the concentrations of free P and free L with given values of K, K_i, and N.

$$[P]_{total} = Z_{PL}[P] + \left(\frac{\partial lnZ_{PD}}{\partial \ln[P]}\right)[D]_{total} = Z_{PL}[P] + \left(\frac{N\sum_{i=1}^{6} K_i[PL_i]}{1+\sum_{i=1}^{6} K_i[PL_i]}\right)[D]_{total} \quad \text{(Eq. S14a)}$$

$$\begin{aligned}[L]_{total} &= [L] + \sum_{i=1}^{6} i[PL_i] + \left(\frac{\partial lnZ_{PD}}{\partial \ln[L]}\right)[D]_{total} \\ &= [L] + \sum_{i=1}^{6} i[PL_i] + \left(\frac{N\sum_{i=1}^{6} iK_i[PL_i]}{1+\sum_{i=1}^{6} K_i[PL_i]}\right)[D]_{total}\end{aligned} \quad \text{(Eq. S14b)}$$

These two polynomials were simultaneously solved by a bracketing method in Igor Pro 8.02 (WaveMatrics). Finally, with the solved values of [P] and [L], the fractional population of n:1 complex between dimeric Zur and DNA was calculated by adding appropriate terms in an expanded form Eq. S13. For instance, the fractional populations of 1:1, 2:1, and 3:1 complexes between dimeric Zur and *zitB* DNA are given by the following equations.

$$f_{1:1} = \frac{3\sum_{i=1}^{6} K_i[PL_i]}{Z_{PD}} \quad \text{(Eq. S15a)}$$

$$f_{2:1} = \frac{3\sum_{i=1}^{6}(K_i[PL_i])^2 + 6\sum_{i=1}^{5}\sum_{j=i+1}^{6} K_iK_j[PL_i][PL_j]}{Z_{PD}} \quad \text{(Eq. S15b)}$$

$$f_{3:1} = \frac{\sum_{i=1}^{6}(K_i[PL_i])^3 + 3\sum_{i=1}^{6}\sum_{j\neq i}^{6}(K_i[PL_i])^2K_j[PL_j] + 6\sum_{i<j<l\leq 6} K_i[PL_i]K_j[PL_j]K_l[PL_l]}{Z_{PD}} \quad \text{(Eq. S15c)}$$

In the simulation, the DNA binding affinity of Zur determined by ITC (Supplementary Table S3) was assigned to K_4 (see Results). On the basis of our previous and current studies (9,10), we deduced that PL_2 is inactive in DNA binding. Hence, we assigned subtle values to K_2 ($\leq 10^4$ M^{-1}) so that there is insignificant fraction of PL_2 bound to DNA at Zur, DNA, and zinc concentrations used in the simulation. The value of K_6 was varied in a wide range ($\geq K_4$). For the values of K_1, K_3, and K_5, we assigned the same or lower values than those of K_2, K_4, and K_6, respectively. Regardless of how these values are assigned, K_6 must be far (about three orders of magnitude) greater than K_4 in order to recapitulate the qualitative features of experimental data (Supplementary Figure S11B and C). The absolute values of the DNA binding affinities affect the breadth of the transition from

dimeric to tetrameric binding of Zur on *znuA* DNA (or tetrameric to hexameric binding on *zitB* DNA) (Supplementary Figure S11B and C).

Because we did not observe any 1:1 complex between dimeric Zur and *zitB* DNA in our EMSA experiments, an alternative model was constructed by subtracting the statistical weight of 1:1 complex from the original DNA binding polynomial.

$$Z_{PD,zitB} = \left(1 + \sum_{i=1}^{6} K_i[PL_i]\right)^N - 3\sum_{i=1}^{6} K_i[PL_i] \quad \text{(Eq. S16)}$$

Then, using the numerical procedure described above, we calculated the fractional populations of the 2:1 and 3:1 complexes as functions of zinc concentration (Supplementary Figure S11D). The simulation is consistent with the conclusion derived from our original model that K_6 must be much greater than K_4 to drive a transition from tetrameric (2:1) binding to hexameric (3:1) binding of Zur on *zitB* DNA.

Although cooperativity between Zur dimers bound to DNA was not explicitly considered in our model, it can be readily incorporated into the model when necessary. For instance, if there is cooperativity between PL_i and PL_j on DNA defined as a factor of ω_{ij}, Eq. S15b and c are modified to include this term.

$$f_{2:1} = \frac{3\sum_{i=1}^{6}(K_i[PL_i])^2\omega_{ii} + 6\sum_{i=1}^{5}\sum_{j=i+1}^{6} K_iK_j[PL_i][PL_j]\omega_{ij}}{Z_{PD}} \quad \text{(Eq. S17a)}$$

$$f_{3:1} = \frac{\sum_{i=1}^{6}(K_i[PL_i])^3\omega_{ii}^3 + 3\sum_{i=1}^{6}\sum_{j\neq i}^{6}(K_i[PL_i])^2K_j[PL_j]\omega_{ii}\omega_{ij}^2 + 6\sum_{i<j<l\leq 6} K_i[PL_i]K_j[PL_j]K_l[PL_l]\omega_{ij}\omega_{jl}\omega_{li}}{Z_{PD}} \quad \text{(Eq. S17b)}$$

Supplementary References

1. Grossoehme, N.E., Spuches, A.M. and Wilcox, D.E. (2010) Application of isothermal titration calorimetry in bioinorganic chemistry. *J Biol Inorg Chem*, **15**, 1183-1191.
2. North, M.L. and Wilcox, D.E. (2019) Shift from Entropic Cu(2+) Binding to Enthalpic Cu(+) Binding Determines the Reduction Thermodynamics of Blue Copper Proteins. *J Am Chem Soc*, **141**, 14329-14339.
3. Fahrni, C.J. and O'Halloran, T.V. (1999) Aqueous Coordination Chemistry of Quinoline-Based Fluorescence Probes for the Biological Chemistry of Zinc. *J Am Chem Soc*, **121**, 11448-11458.
4. Microcal, I. (2004) ITC Data Analysis in Origin. *ITC Data Analysis in Origin*, 104-106.
5. Eftink, M.R., Anusiem, A.C. and Biltonen, R.L. (1983) Enthalpy-entropy compensation and heat capacity changes for protein-ligand interactions: general thermodynamic models and data for the binding of nucleotides to ribonuclease A. *Biochemistry*, **22**, 3884-3896.
6. Grossoehme, N.E. and Giedroc, D.P. (2009) Energetics of allosteric negative coupling in the zinc sensor S. aureus CzrA. *J Am Chem Soc*, **131**, 17860-17870.
7. Rich, A.M., Bombarda, E., Schenk, A.D., Lee, P.E., Cox, E.H., Spuches, A.M., Hudson, L.D., Kieffer, B. and Wilcox, D.E. (2012) Thermodynamics of Zn2+ binding to Cys2His2 and Cys2HisCys zinc fingers and a Cys4 transcription factor site. *J Am Chem Soc*, **134**, 10405-10418.
8. Kozlov, A.G. and Lohman, T.M. (2000) Large contributions of coupled protonation equilibria to the observed enthalpy and heat capacity changes for ssDNA binding to Escherichia coli SSB protein. *Proteins*, **Suppl 4**, 8-22.
9. Choi, S.H., Lee, K.L., Shin, J.H., Cho, Y.B., Cha, S.S. and Roe, J.H. (2017) Zinc-dependent regulation of zinc import and export genes by Zur. *Nat Commun*, **8**, 15812.
10. Shin, J.H., Jung, H.J., An, Y.J., Cho, Y.B., Cha, S.S. and Roe, J.H. (2011) Graded

expression of zinc-responsive genes through two regulatory zinc-binding sites in Zur. *Proc Natl Acad Sci U S A*, **108**, 5045-5050.

11. Gilston, B.A., Wang, S., Marcus, M.D., Canalizo-Hernandez, M.A., Swindell, E.P., Xue, Y., Mondragon, A. and O'Halloran, T.V. (2014) Structural and mechanistic basis of zinc regulation across the E. coli Zur regulon. *PLoS Biol*, **12**, e1001987.
12. Yang, X., Wang, Y., Liu, G., Deng, Z., Lin, S. and Zheng, J. (2022) Structural basis of Streptomyces transcription activation by zinc uptake regulator. *Nucleic Acids Res*, **50**, 8363-8376.
13. Higgins, N.P. (2005) *The Bacterial Chromosome*. ASM Press, Washington, DC.
14. Holbrook, J.A., Tsodikov, O.V., Saecker, R.M. and Record, M.T., Jr. (2001) Specific and non-specific interactions of integration host factor with DNA: thermodynamic evidence for disruption of multiple IHF surface salt-bridges coupled to DNA binding. *J Mol Biol*, **310**, 379-401.
15. Koh, J., Saecker, R.M. and Record, M.T., Jr. (2008) DNA binding mode transitions of Escherichia coli HU(alphabeta): evidence for formation of a bent DNA--protein complex on intact, linear duplex DNA. *J Mol Biol*, **383**, 324-346.

Supplementary Tables

Table S1. Zinc content of purified apo-Zur measured by ICP-MS

Sample	$[Zn^{2+}]$ / [Zur monomer]
Sample 1	0.93
Sample 2	1.02
Sample 3	0.94

Table S2. Thermodynamics of the zinc binding of *Sc*Zur determined by ITC[a]

		N (sites)	*K* (M^{-1})	$\Delta H°$ (kcal/mol)	$T\Delta S°$ (kcal/mol)
WT Zur	Apparent[b]	6.2 ± 0.1	8.6 (± 1.9) x 10^5 (1.2 ± 0.3 μM)[d]	3.7 ± 0.6	11.8 ± 0.6
	Intrinsic[c]	6.2 ± 0.1	2.3 (± 0.5) x 10^{13} (44.2 ± 9.9 fM)[d]	- 9.3 ± 0.6	8.9 ± 0.6
H36A	Apparent[b]	4.0 ± 0.1	9.5 (± 2.7) x 10^5 (1.1 ± 0.3 μM)[d]	3.0 ± 0.3	11.2 ± 0.4
	Intrinsic[c]	4.0 ± 0.1	2.5 (± 0.7) x 10^{13} (40.2 ± 11.3 fM)[d]	- 10.0 ± 0.3	8.3 ± 0.4
H84AE105A	Apparent[b]	4.0 ± 0.1	9.5 (± 3.4) x 10^5 (1.1 ± 0.4 μM)[d]	4.2 ± 0.3	12.3 ± 0.4
	Intrinsic[c]	4.0 ± 0.1	2.5 (± 0.9) x 10^{13} (40.3 ± 14.5 fM)[d]	- 8.9 ± 0.3	9.4 ± 0.4
C79SH87A	Apparent[b]	2.6 ± 0.3	2.9 (± 1.5) x 10^6 (0.35 ± 0.18 μM)[d]	4.1 ± 0.3	12.9 ± 0.5
	Intrinsic[c]	2.6 ± 0.3	7.7 (± 4.0) x 10^{13} (13.0 ± 6.7 fM)[d]	- 8.9 ± 0.3	10.0 ± 0.5

a. The errors are the standard deviations of the best-fit parameters obtained from multiple (≥ 3) ITC experiments.

b. ITC data were analyzed by a single-class site model to obtain apparent thermodynamic parameters.

c. ITC data were analyzed by a competition model to obtain intrinsic thermodynamic parameters (Supplementary Information S1).

d. Dissociation constant (1/K)

Table S3. Thermodynamics of the DNA binding of *Sc*Zur determined by ITC[a]

		N (sites)	*K* (M^{-1})	$\Delta H°$ (kcal/mol)	$T\Delta S°$ (kcal/mol)
WT Zur	*znuA*	2.1 ± 0.1	8.7 (± 1.2) x 10^5 (1.2 ± 0.2 μM)[b]	- 11.8 ± 2.6	- 3.7 ± 2.6
	zitB	3.1 ± 0.1	1.8 (± 0.2) x 10^6 (0.6 ± 0.1 μM)[b]	- 6.9 ± 0.8	1.6 ± 0.8
H36A	*znuA*	2.0 ± 0.2	3.3 (± 1.0) x 10^5 (3.0 ± 0.9 μM)[b]	- 18.4 ± 3.9	- 10.8 ± 3.9
	zitB	3.1 ± 0.1	2.6 (± 0.7) x 10^5 (3.9 ± 1.0 μM)[b]	- 19.5 ± 5.4	- 12.1 ± 5.4

a. ITC data were analyzed by a single-class site model. The errors are the standard deviations of the best-fit parameters obtained from multiple (≥ 3) ITC experiments.

b. Dissociation constant (1/K)

Table S4. Comparison between direct and *post hoc* methods correcting the apparent zinc binding parameters of Zur for the EGTA competition effect

		K (M^{-1})	$\Delta H°$ (kcal/mol)
WT Zur	Direct	2.3 (± 0.5) x 10^{13}	- 9.3 ± 0.6
	Post hoc	2.3 (± 0.5) x 10^{13}	- 9.3 ± 0.6
H36A	Direct	2.5 (± 0.7) x 10^{13}	- 10.0 ± 0.3
	Post hoc	2.5 (± 0.7) x 10^{13}	- 10.0 ± 0.3
H84AE105A	Direct	2.5 (± 0.9) x 10^{13}	- 8.9 ± 0.3
	Post hoc	2.5 (± 0.9) x 10^{13}	- 8.9 ± 0.3
C79SH87A	Direct	7.7 (± 4.0) x 10^{13}	- 8.9 ± 0.3
	Post hoc	7.6 (± 4.0) x 10^{13}	- 8.9 ± 0.3

Table S5. Bacterial strains used in this study.

Strains	Genotype or description	Reference or sources
DH5**α**	supeE44, ΔlacU169 (φ80lacZΔM15), hsdr17 (rK-, mK-), recA1, endA1, gy rA96, thi-1, relA1	Laboratory Stock
BL21(DE3)pLysS	F^- *ompT* r_B^- m_B^- (DE3)/pLysS	Laboratory Stock
ET12567 (pUZ80 02)	F^- *dam13*::Tn9 *dcm6 hsdM hsdR recF143*::Tn10 *galK2 galT22 ara-1 4 lacY1 xyl-5 leuB6 thi-1 tonA31 r psL136 hisG4 tsx-78 mtl-1 glnV44*	Laboratory Stock
M145	Prototrophic $SCP1^-SCP2^-Pgl^+$	Laboratory Stock
S700	M145 *zur*::Apr^R	(10)
S162	M145 *pSET162*::Apra/Tsr^R	(10)
S706	S700+*pSET162zur*WT::Apra/Tsr^R	(10)
E25A	S700+*pSET162zur*E25A::Apra/Tsr^R	This study
E27A	S700+*pSET162zur*E27A::Apra/Tsr^R	This study
E28A	S700+*pSET162zur*E28A::Apra/Tsr^R	(10)
E34A	S700+*pSET162zur*E34A::Apra/Tsr^R	This study
H36A	S700+*pSET162zur*H36A::Apra/Tsr^R	(10)
D37A	S700+*pSET162zur*D37A::Apra/Tsr^R	This study
H41A	S700+*pSET162zur*H41A::Apra/Tsr^R	(10)
D44A	S700+*pSET162zur*D44A::Apra/Tsr^R	This study
D60A	S700+*pSET162zur*D60A::Apra/Tsr^R	This study

Table S6. Plasmids used in this study.

Plasmids	Description	Reference or Sources
pET3a	An overexpression plasmid carrying a mpicillin resistance marker and T7 pr omoter	Laboratory Stock
pSET162	A derivative of integrative plasmid pS ET151 carrying a thiostrepton resistan ce marker	Laboratory Stock
PSJ703	A derivative of pGEM-Teasy carrying a *zur* wild type gene	(10)

Table S7. Oligonucleotides used in this study

Name	Sequence (5' to 3')
**Site specific mutagenesis of zur**	
Zur F[a]	TTTCATATGACCACCGCTGGACCGCCC
Zur R[b]	TTTGGATCCTCAACCGCCGGAGGCCCCCG
Zur E25A F	GCCCTTCAGGCGGTCGAGGAGTTC
Zur E25A R	TTTGGATCCTCAACCGCCGGAGGCCCCCG
Zur E27A F	CTTCAGGAGGTCGCGGAGTTC
Zur E27A R	GAACTCCGCGACCTCCTGAAG
Zur E28A F	CTTCAGGAGGTCGCGGCGTTC
Zur E28A R	GAA CGC CTC GAC CTC CTG AAG
Zur E34A F	CGCAGGCGCTCCACGACATG
Zur E34A R	CATGTCGTGGAGCGCCTGCG
Zur H36A F	CAGGAGCTCGCAGACATGCTC
Zur H36A R	GAGCATGTCTGCGAGCTCCTG
Zur D37A F	GGAGCTCCACGCCATGCTCAAGC
Zur D37A R	GCTTGAGCATGGCGTGGAGCTCC
Zur H41A F	GATGCTCAAGGGCAAGGGCGAC
Zur H41A R	GTCGCCCTTGCCCTTGAGCATC
Zur D44A F	GCTCAAGCACAAGGGCGCCG
Zur D44A R	CGGCGCCCTTGTGCTTGAGC
Zur D60A F	CCTTCAGTCCCTCGCCGCCG
Zur D60A R	CGGCGGCGAGGGACTGAAGG
Zur C79SH87A F	CATCACCACGCCCTGGTCTGC
Zur C79SH87A R	GCAGACCAGGGCGTGGTGATG
Zur H84AE105A F	CACCGGCGACGCTCACCACCAC
Zur H84AE105A R	GTGGTGGTGAGCGTCGCCGGTG
**RT PCR**	
znuA RT F	GAACGTACGACGACGCCGCA
znuA RT R	CCGAGCTGTCGCTGGAGCAG
zitB RT F	GGTACACACGAGGAGGCGGC
zitB RT R	GTGATCGACAGCGCCACGCG
16S RT F	GAGTTGCAGACCCCAATCCG
16S RT R	TGCCCCTTATGTCTTGGGCT
**S1 probe**	
zitB S1 F	GTCGGACCGGTCCCCCTGAC
zitB S1 R	GGGTGATCGACAGCGCCACG
**DNA binding experiment**	
znuA 33 bp F	TCCGCTTGTTGACAACGGTTTCCATATTTGTTG
znuA 33 bp R	CAACAAATATGGAAACCGTTGTCAACAAGCGGA
zitB 33 bp F	CAAGGCACATGACAACGGTGTTCAGTGCCGCGT
zitB 33 bp R	ACGCGGCACTGAACACCGTTGTCATGTGCCTTG
znuA zurbox with zitB flank regions F	TCCGCCACATGACAACGGTTTCCAGTGCTGTTG
znuA zurbox with zitB flank regions R	CAACAGCACTGGAAACCGTTGTCATGTGGCGGA

zitB zurbox with znuA flank regions F	CAAGGTTGTTGACAACGGTGTTCATATTCGCGT
zitB zurbox with znuA flank regions R	ACGCGAATATGAACACCGTTGTCAACAACCTTG
zitB footprinting probe F	GACAAACCGCGCCCCCAGA
zitB footprinting probe R	CTCCTCGTGTGTACCGGCGTC

a: *NdeI* site is underlined.

b: *BamHI* site is underlined.

Supplementary Figures

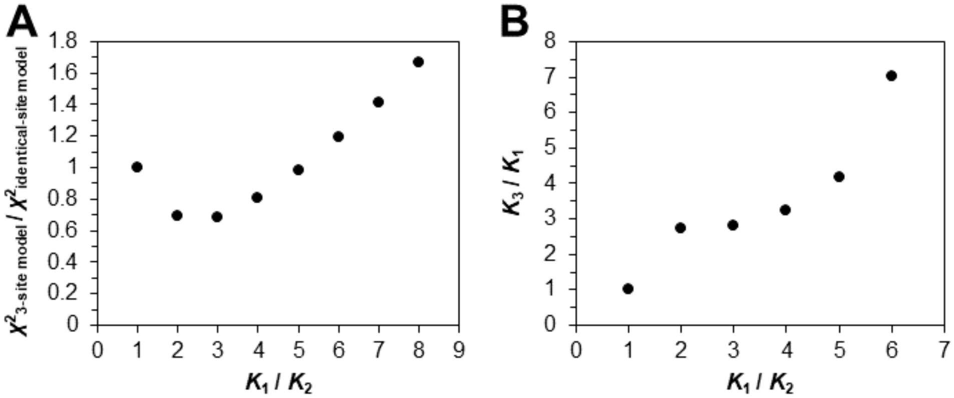

Supplementary Figure S1. Analysis of ITC data for the Zur-zinc interaction by a three-site model to probe potential heterogeneity among the three zinc binding sites in Zur. Because the ITC curve is apparently monophasic (Figure 1), it allows unique determination of only a single set of K and ΔH. Thus, K_1 was first fixed at the value obtained from the identical site model (Table S2). Then, K_2 was systematically decreased from the K_1 value while K_3 was floated in the fitting procedure. Likewise, ΔH_1 was fixed at the fit-value of the identical site model while ΔH_2 was increased by $-RT\ln(K_2/K_1)$. Finally, ΔH_3 was floated, together with K_3, to fit the ITC data to the three-site model. Up to a 5-fold change in K_2 yielded the fitting quality (judged by the χ^2 value) better than or comparable to the identical site model (i.e., χ^2 ratio ≤ 1) (A). In this range of K_2, the best-fit value of K_3 increased from K_1 to $4.2K_1$ (B). Further decreasing K_2 deteriorated the fitting quality (A). Therefore, the maximum of 20-fold difference (K_3/K_2) between zinc binding sites in Zur can be tolerated by our ITC data.

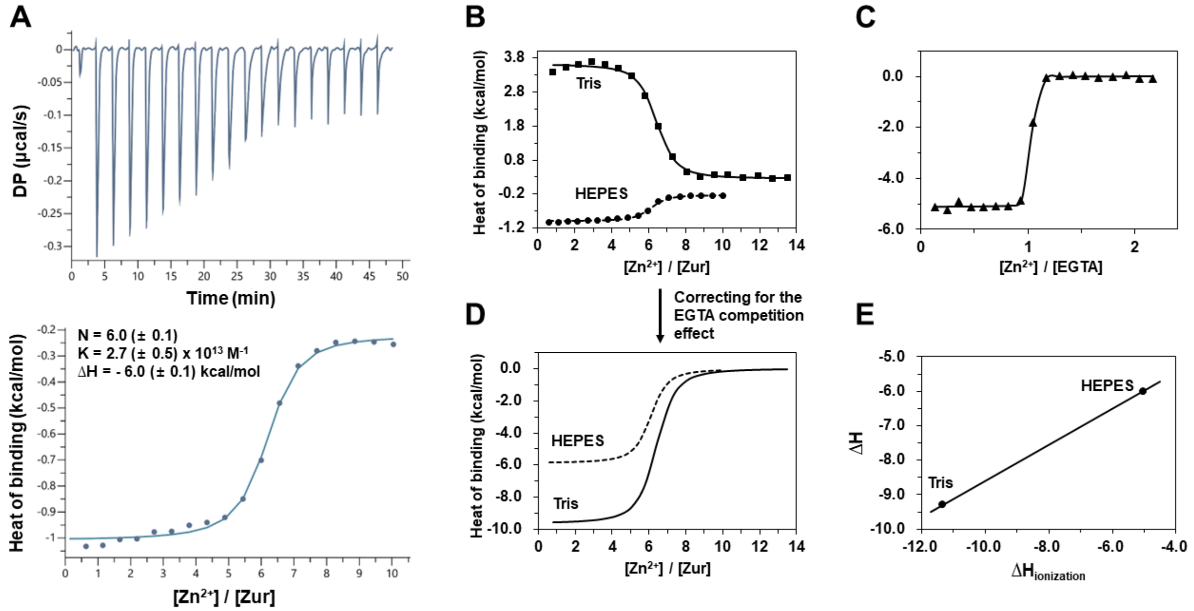

Supplementary Figure S2. Probing potential effects of buffer ionization enthalpy on the ITC curve for the Zur-zinc interaction. (A) Zinc (2.6 mM $ZnCl_2$) was titrated into 50 µM dimeric apo-Zur in the binding buffer containing 20 mM HEPES (pH 7.8), 150 mM NaCl, 0.5 mM TCEP, and 4.0 mM EGTA at 25 °C. At this condition, the ITC curve is monophasic even though the ionization enthalpy of HEPES is much smaller in magnitude than that of Tris. (B) The difference in buffer ionization enthalpy results in opposite signs of the Zur-zinc binding heat between Tris and HEPES buffers. In titrating zinc into Zur in the presence of the competitor EGTA, buffer is deprotonated upon dissociation of zinc from EGTA but protonated upon association of zinc with Zur. Therefore, the net change in protonation state of buffer and the magnitude of buffer ionization enthalpy determine the difference in overall binding heat between the two buffers. The ITC curve was first fitted to Eq. S6-9 accounting for the EGTA competition effect. The fitting procedure utilized the EGTA-zinc binding enthalpy (inherently including the accompanying buffer ionization heat) determined by an independent ITC measurement (C) and the NIST approved binding affinity at pH 7.8 (The EGTA-zinc titration in Tris buffer is shown in Supplementary Figure S13). This analysis yielded the Zur-zinc binding stoichiometry and affinity (N and K shown

in (A)) consistent with those quantified in Tris buffer (Table S2) within experimental uncertainty. The best-fit Zur-zinc binding enthalpy is different between Tris and HEPES buffers and the difference is reflected in the ITC curves corrected for the EGTA competition effect (D). (E) From the two binding enthalpies measured in Tris and HEPES buffers, the buffer-independent binding enthalpy (ΔH_{ind} = – 3.4 kcal/mol) and the number of protons released per zinc-binding site in Zur (Δn_H = 0.5) were estimated by using the relationship $\Delta H = \Delta H_{ind} + \Delta n_H(\Delta H_{buffer\text{-}ionization})$.

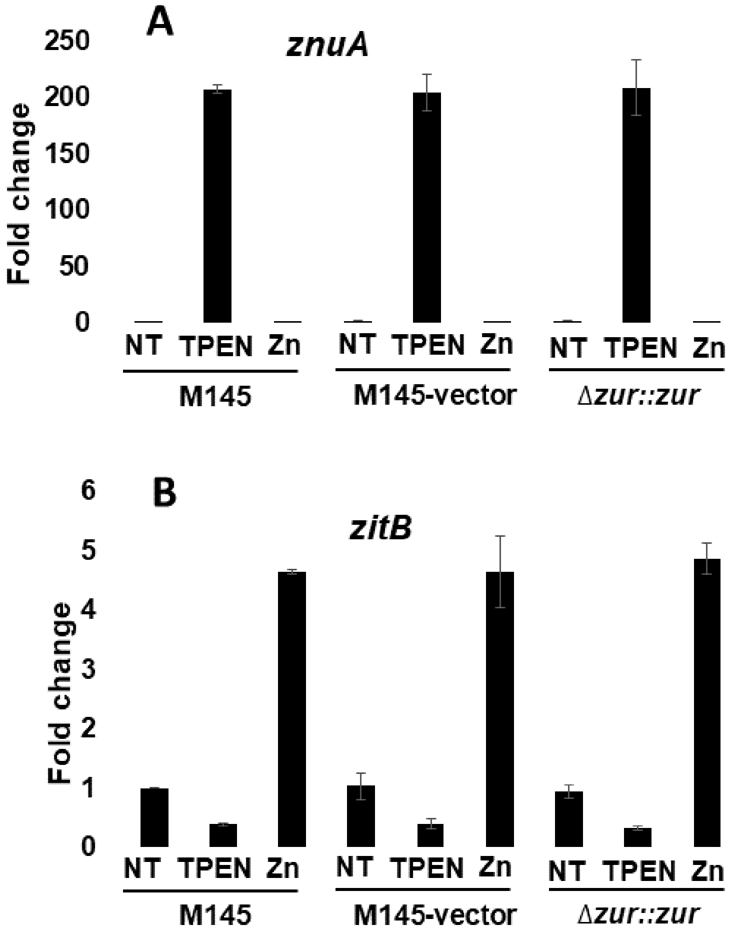

Supplementary Figure S3. Comparison of *znuA* and *zitB* expression patterns of M145, M145-vector, and *Δzur::zur*. Transcript levels of *znuA* (A) and *zitB* (B) of M145, M145-vector, and *Δzur::zur* cells were determined by qRT-PCR. Error bars were presented from the three independent experiments.

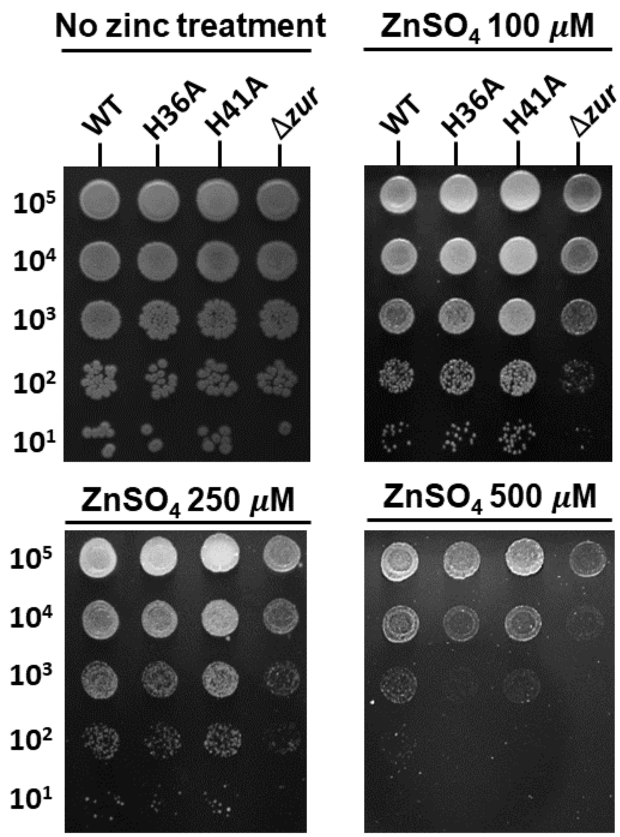

Supplementary Figure S4. Sensitivity of *S.coilcolor* to high zinc stress. The sensitivity of wild-type (Δ*zur::zur*), H36A, H41A, and Δ*zur* cells to high zinc stress was assessed by a spotting assay. From 10 to 10^5 cells were spotted on nutrient agar (NA) plates, and incubated for 48 hr at 30 ℃.

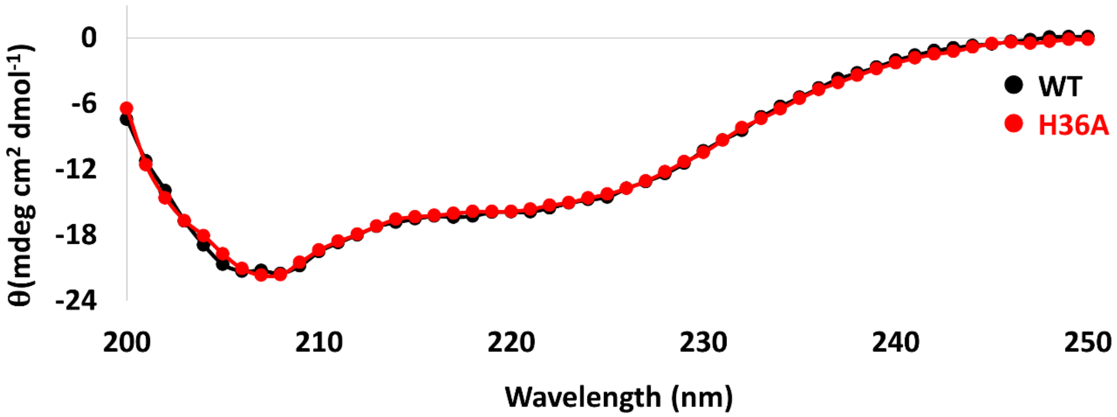

Supplementary Figure S5. Comparison between the circular dichroism spectra of wild-type and H36A apo-Zur. Approximately 20 μM of wild-type (black) and H36A Zur (red) dimer were used to measure circular dichroism spectra.

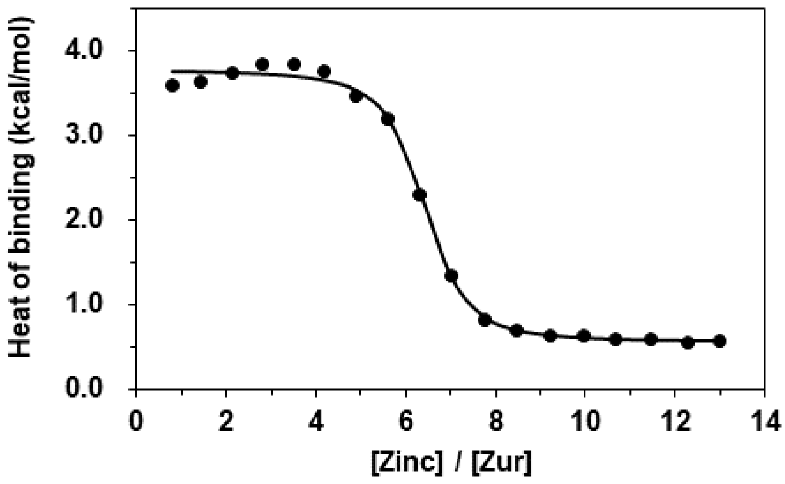

Supplementary Figure S6. Analysis of zinc binding to H41A Zur by ITC. Zinc (2.0 mM $ZnCl_2$) was titrated into dimeric apo-Zur of the H41A mutant (30 μM) in the binding buffer at 25 °C. The ITC curve was fitted to a single-class site model (solid line), yielding the binding stoichiometry (n = 6.1 ± 0.1), affinity (K = 2.7 (± 0.5) x 10^{13} M^{-1}) and enthalpy ($\Delta H°$ = - 9.8 ± 0.1 kcal/mol), all of which were consistent with those of the wild type (Table S2) within experimental uncertainty.

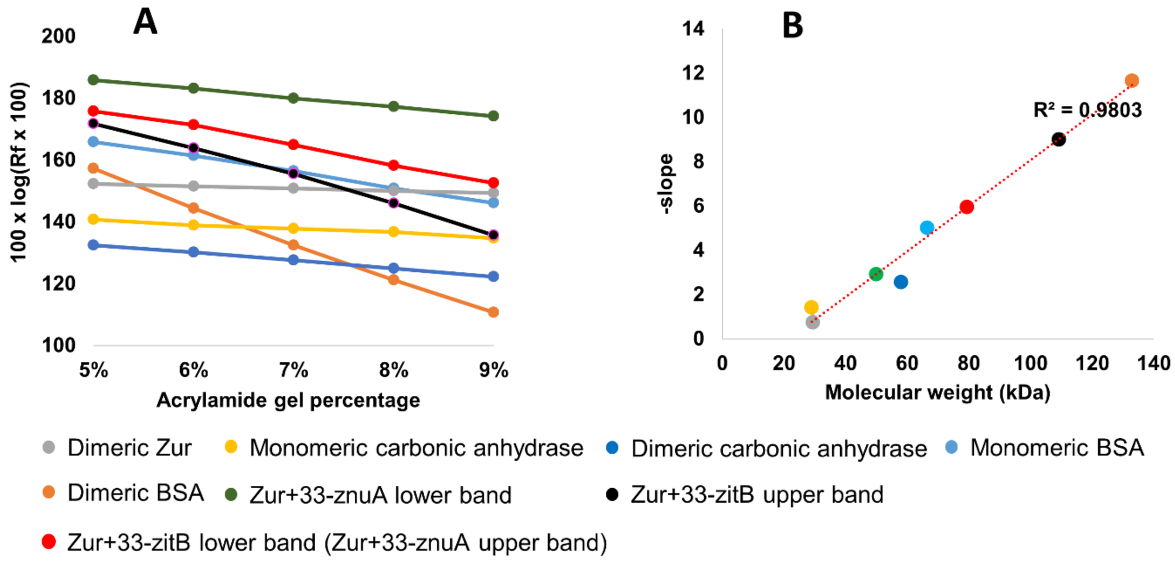

Supplementary Figure S7. Measurement of the stoichiometries of Zur-*zitB* DNA complexes by native PAGE mobility. (A) Relative mobilities of Zur-33 bp *zitB* DNA probe on native PAGE on 5 to 9% polyacrylamide gels. Logarithms of relative mobilities of Zur-DNA complexes and standard proteins were plotted against acrylamide percentage as described previously (9). For standard proteins, BSA monomer (sky blue), BSA dimer (orange), carbonic anhydrase dimer (blue), Zur dimer (gray) were used. (B) Determination of the molecular weights and stoichiometries of Zur-*zitB* DNA complexes. A standard curve was obtained by re-plotting the negative slopes of the mobility lines for each standard proteins against their molecular weight using the least squares regression. The well-fitting line (R^2 of 0.9803) was obtained when the lower and upper bands on 33 bp *zitB* DNA probes were assumed as tetrameric (79.43 kDa, red) and hexameric (109.1 kDa, black) Zur-bound complexes, respectively.

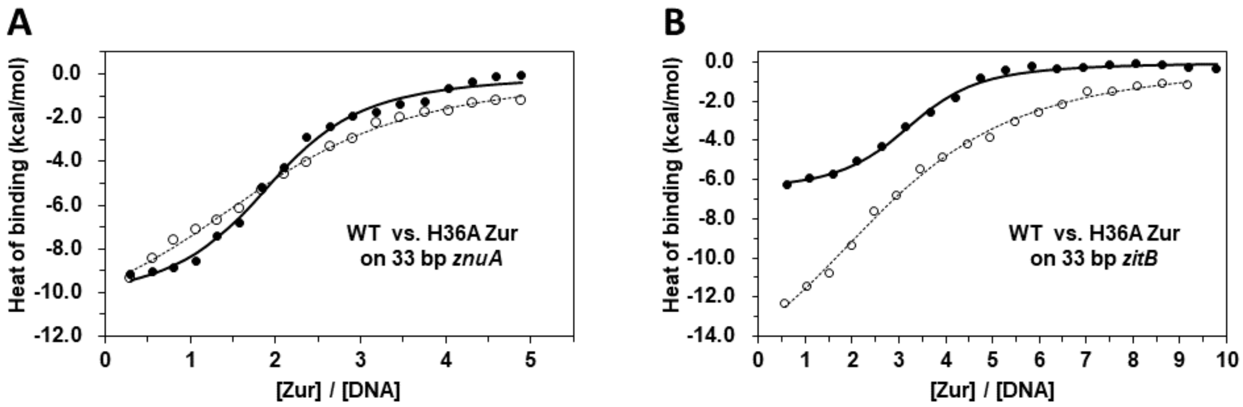

Supplementary Figure S8. Comparison between WT and H36A Zur in binding to the specific DNA probes. (A) ITC curves (reproduced from Figure 6) are shown for the binding of WT (closed circles) and H36A (open circles) to the 33 bp *znuA* DNA probe with the corresponding fits to a single-class site model. (B) ITC curves are shown for the binding of WT (closed circles) and H36A (open circles) to the 33 bp *zitB* DNA probe with the corresponding fits to a single-class site model. Note that the slope near the inflection point is steeper (i.e., higher affinity) for WT than for H36A even though the binding heat signal is larger in magnitude for H36A.

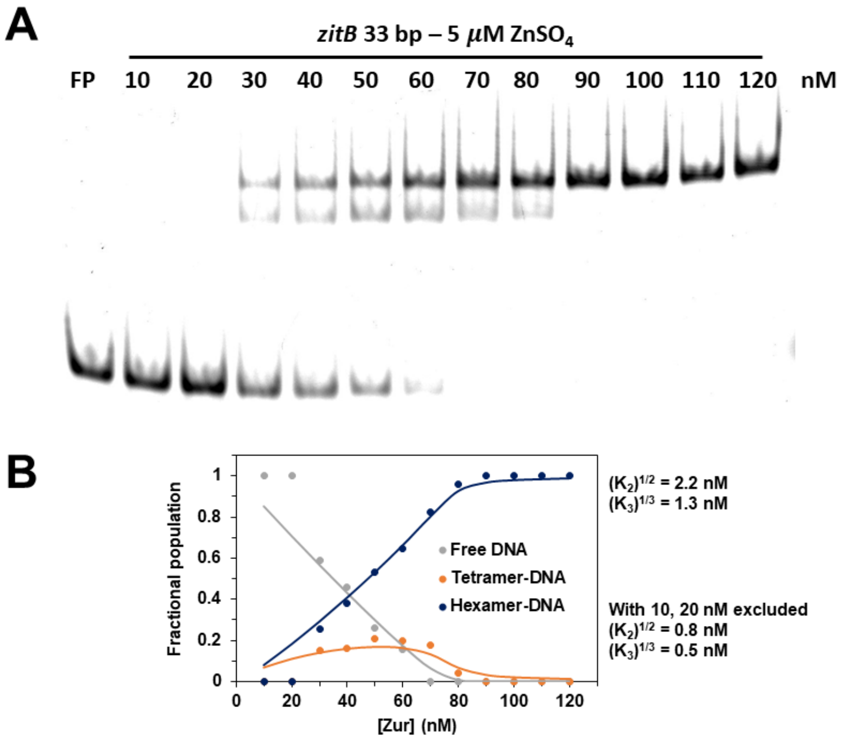

Supplementary Figure S9. Measurement of the DNA binding affinity of zinc-saturated Zur. (A) Increasing amount of Zur was incubated with the FAM-labeled *zitB* DNA probe in the presence of the excess amount of $ZnSO_4$. The retarded bands corresponding to tetrameric and hexameric complexes were detected in native PAGE. (B) Fractional distribution among free (D_f), tetrameric (D_t), and hexameric Zur-bound DNA (D_h) was calculated from the relative band intensities shown in (A). The distribution was analyzed to determine the binding constants K_2 and K_3 for formation of the 2:1 and the 3:1 complex, respectively. In brief, D_f, D_t, and D_h are given by $\frac{1}{1+K_2[Zur]^2+K_3[Zur]^3}$, $\frac{K_2[Zur]^2}{1+K_2[Zur]^2+K_3[Zur]^3}$, and $\frac{K_3[Zur]^3}{1+K_2[Zur]^2+K_3[Zur]^3}$, respectively, where [Zur] is the concentration of DNA-unbound Zur (but saturated with zinc). [Zur] can be calculated for given values of K_2 and K_3 by numerically solving the mass balance equation $[Zur]_{tot} = [Zur] + \frac{2K_2[Zur]^2}{1+K_2[Zur]^2+K_3[Zur]^3}[DNA]_{tot} + \frac{3K_3[Zur]^3}{1+K_2[Zur]^2+K_3[Zur]^3}[DNA]_{tot}$ where $[Zur]_{tot}$ and $[DNA]_{tot}$ are the total concentrations of Zur

and DNA, respectively, at a given titration point. Then, a set of K_2 and K_3 yielding D_f, D_t, and D_h which collectively best fit the EMSA-derived population distribution was obtained by the nonlinear least-squares minimization method in Igor Pro 8.02 (WaveMatrics) utilizing the Levenberg-Marquadt algorithm. The best-fit DNA binding constants, expressed as per-site affinity ($(K_2)^{1/2}$ and $(K_3)^{1/3}$), were in the low nanomolar range (B). The fitted population curve was of excellent quality except for the first two data points (B), which possibly undermined the quality, since adsorption of protein to a sample vial aggravates typically at very low protein concentrations. Analysis of the population distribution excluding these data points yielded slightly higher (sub-nanomolar) binding constants.

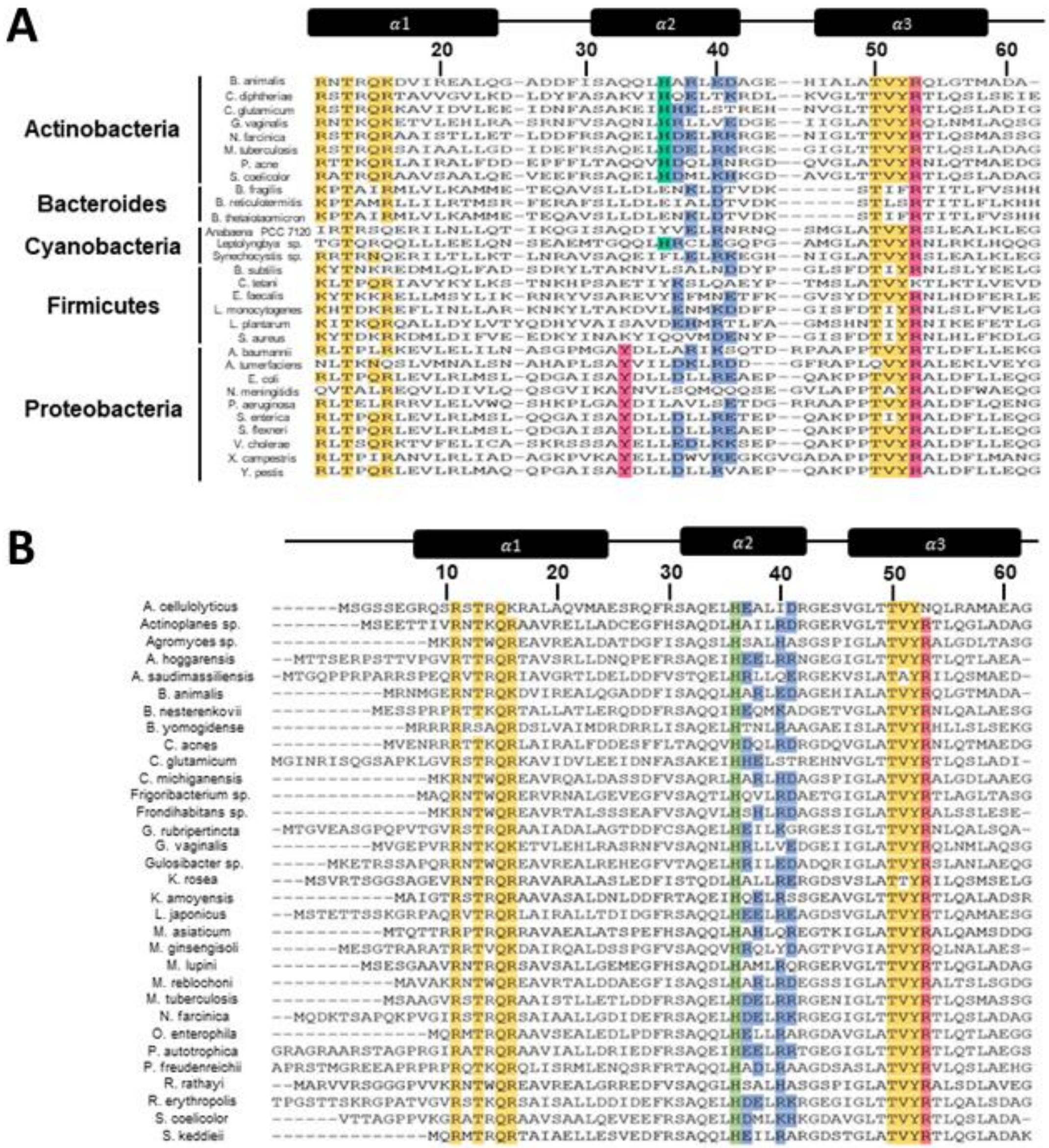

Supplementary Figure S10. Conservation of H36 across bacterial phyla. Multiple sequence alignment of DNA binding domains of (A) 29 selected Zur proteins from different bacterial phyla and (B) 32 selected Actinobacterial Zur were performed by Clustal W program. Conserved residues corresponding to those involved in DNA recognition in *E. coli* Zur-znuA (11) and *S. coelicolor* Zur-zitB (12) interactions were highlighted in different colors (Red for base-specific interaction, yellow for highly conserved DNA binding

residues, and blue for salt bridge formation). The histidines corresponding to H36 of *S. coelicolor* Zur were highlighted in green.

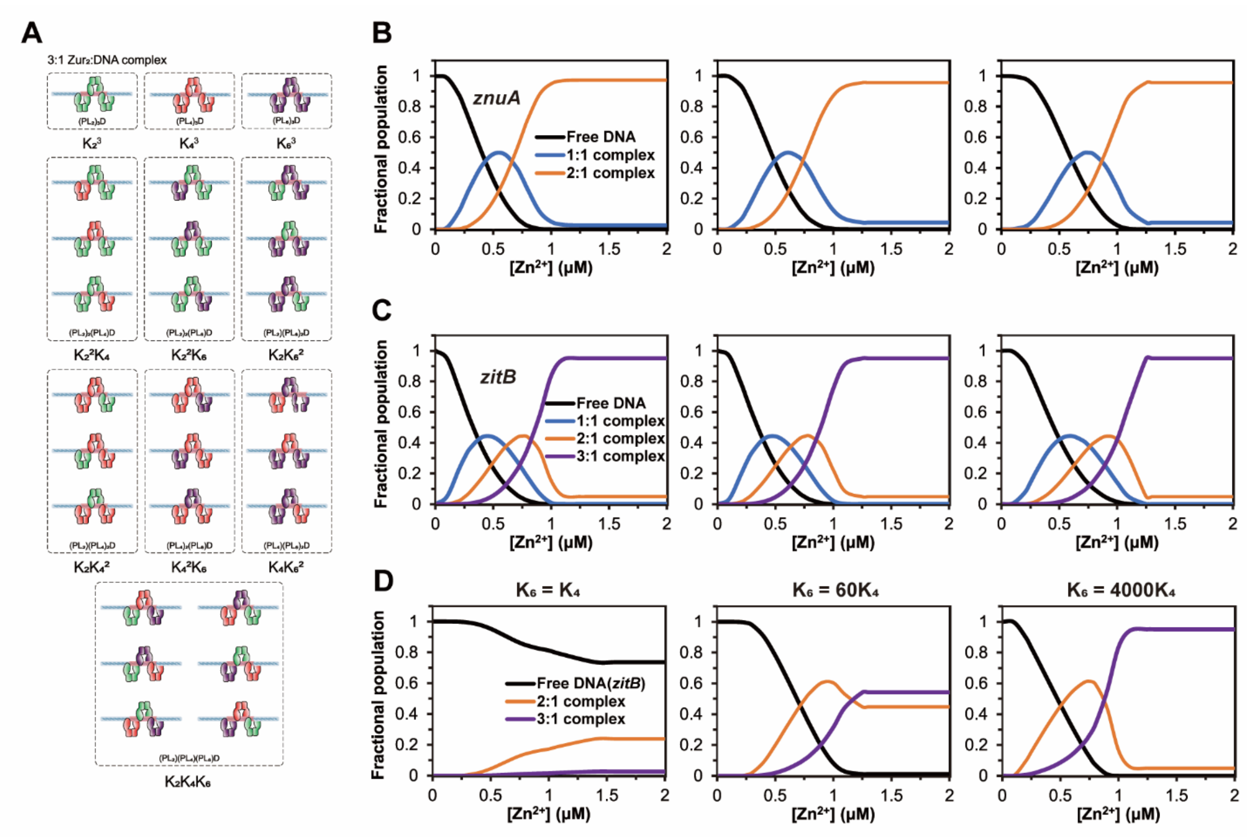

Supplementary Figure S11. Thermodynamic model for coupling between the zinc binding and DNA binding equilibria of Zur. (A) Schematic illustration for a full set of molecular states of the 3:1 (Zur dimer:*zitB* DNA) complex with various combinations of PL_i bound to DNA (see Figure 7A for the entire equilibrium illustration). (B and C) Population distribution among free DNA (B: *znuA*; C: *zitB*), 1:1, 2:1, and 3:1 complexes as a function of zinc concentration for various assignments of K_3 and K_5. The first panels are the same as the third panels of Figure 7B (K_6 = 700K_4) and C (K_6 = 4000K_4) where $K_5 = K_6$ and $K_3 = K_4$. In the second panels, K_5 and K_3 were assumed to be half the values of K_6 and K_4, respectively ($K_5 = 0.5K_6$ and $K_3 = 0.5K_4$). In the third panels, the zinc binding free energy ($\Delta G° = – RT \ln K$, R = 1.99 cal/mol/K, T = 298 K) was assumed to increase in magnitude linearly with the number of zinc ions bound to Zur. Thus, $\Delta G_5° = (\Delta G_6° + \Delta G_4°)/2$ and $\Delta G_3° = (\Delta G_4° + \Delta G_2°)/2$. In turn, $K_5 = e^{-\Delta G_5°/RT}$ and $K_3 = e^{-\Delta G_3°/RT}$. In all cases, because K_6 is much greater than K_4, the transition from dimeric to tetrameric binding of

Zur on *znuA* DNA or from tetrameric to hexameric binding of Zur on *zitB* DNA was successfully simulated. The absolute values of the DNA binding affinities affect the half-transition points (i.e., zinc concentrations where [1:1 complex] = [2:1 complex] on *znuA* DNA or [2:1 complex] = [3:1 complex] on *zitB* DNA). (D) Population distribution among free DNA (*zitB*), 2:1, and 3:1 complexes as a function of zinc concentration. In this simulation, the 1:1 complex was excluded from the original coupling model to account for the EMSA results showing the 2:1 and 3:1 complexes only.

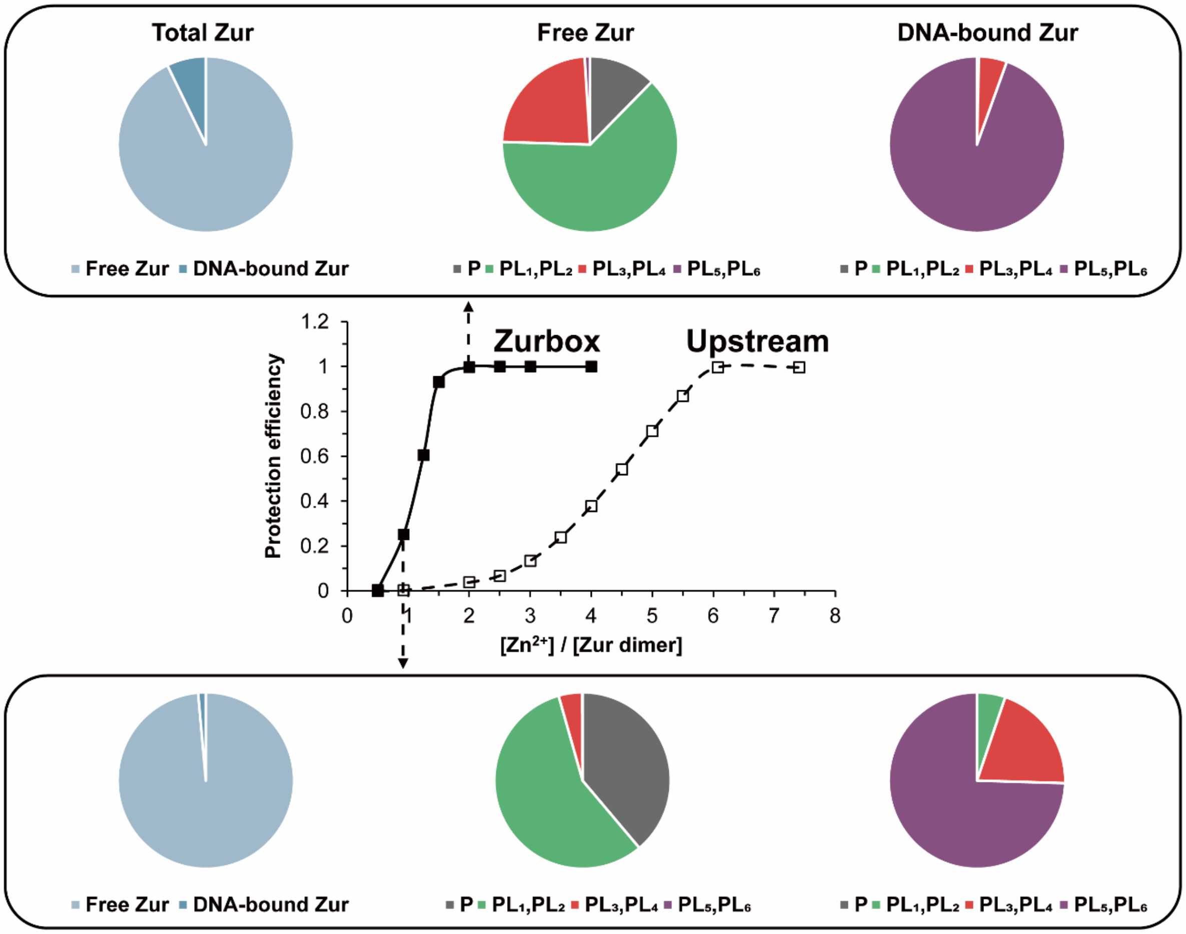

Supplementary Figure S12. Simulation of the sequential protection of the consensus zurbox and the upstream region of the *zitB* gene by Zur by modifying the thermodynamic model to include multimeric binding of Zur to the *zitB* upstream. In this simulation, we assumed the presence of 20 identical nonspecific binding sites in the upstream region for simplicity, and used the DNA binding affinities of 1 μM and 10 μM for $PL_{5\text{ or }6}$ and $PL_{3\text{ or }4}$, respectively. These affinities are within a range observed for nonspecific DNA binding of nucleoid associated proteins present at micromolar concentrations in bacteria (13-15). The protection efficiency was defined as fraction of DNA site (zurbox or upstream site) bound to Zur. Initially, at low zinc concentrations, free Zur would be partially occupied by zinc at the regulatory sites. However, in the population of DNA-bound Zur, the zinc binding

equilibrium would substantially shift toward the high-occupancy state due to its high affinity for the zurbox and to the excess amount of Zur over DNA ([Zur dimer]/[DNA] ~ 40). In addition, because of such an excess amount, only a small fraction of Zur is required to saturate the zurbox. Our simulation shows that full protection of the zurbox is indeed achieved by less than 10 % of the entire Zur population at $[Zn^{2+}]$/[Zur dimer] = 2. Furthermore, at this condition, while Zur with one regulatory site occupied (PL_1, PL_2) is predominant in the DNA-unbound portion of Zur, the majority of the DNA-bound population comprises Zur with three regulatory sites occupied (PL_5, PL_6). On the other hand, the upstream DNA is out-competed for Zur binding and left mostly unprotected during the saturation of the zurbox. However, as the zinc concentration is further increased, the entire population of Zur becomes saturated with zinc and effectively binds the upstream DNA, producing the maximum protection of this region at $[Zn^{2+}]$/[Zur dimer] ≥ 6.

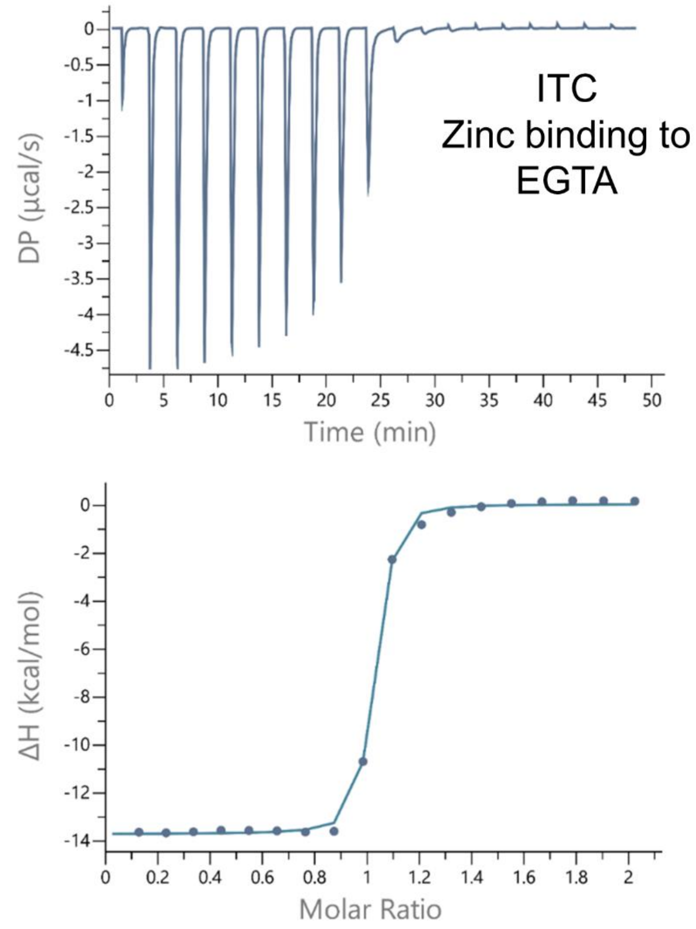

Supplementary Figure S13. ITC measurement of the zinc binding enthalpy of EGTA. 1.95 mM zinc was titrated into 200 μM EGTA in the binding buffer containing 20 mM Tris, pH 7.8, 150 mM NaCl, and 0.5 mM TCEP at 25 °C. The upper panel shows representative ITC heat signals generated in the titration as time traces. The raw heat signals were integrated over time and normalized per mol of zinc injected to obtain heats of binding plotted in the bottom panel as a function of molar ratio [Zn^{2+}]/[EGTA]. The normalized ITC curve was fitted to a single-class site model (continuous line), yielding the binding stoichiometry, affinity, and enthalpy. However, a high c-value (K[EGTA]$_{total}$ >> 1000) precluded reliable affinity measurement while the other two interaction parameters were precisely determined.
